# Impact of Early-Life Brain Injury on Gut Microbiota Composition in Rodents: Systematic Review with Implications for Neurodevelopment

**DOI:** 10.3390/cells14141063

**Published:** 2025-07-11

**Authors:** Vanessa da Silva Souza, Raul Manhães-de-Castro, Sabrina da Conceição Pereira, Beatriz Souza de Silveira, Caio Matheus Santos da Silva Calado, Henrique José Cavalcanti Bezerra Gouveia, Jacques-Olivier Coq, Ana Elisa Toscano

**Affiliations:** 1 Graduate Program in Neuropsychiatry and Behavioral Sciences, Center for Medical Sciences, Federal University of Pernambuco, Recife 50670-901, Pernambuco, Brazil; ft.vanessasouza@gmail.com (V.d.S.S.); beatriz.silveira@ufpe.br (B.S.d.S.); caio.matheus@ufpe.br (C.M.S.d.S.C.); 2 Studies in Nutrition and Phenotypic Plasticity Unit, Center for Health Sciences, Federal University of Pernambuco, Recife 50670-420, Pernambuco, Brazil; raulmanhaesdecastro@gmail.com (R.M.-d.-C.); ft.sabrinapereira@gmail.com (S.d.C.P.); henriquegouveia.93@hotmail.com (H.J.C.B.G.); 3 Institut des Sciences du Mouvement (ISM), Equipe DynamiCC, UMR 7287 CNRS/Aix Marseille Université, 13000 Marseille, France; jacques-olivier.coq@cnrs.fr; 4 Nursing Unit, Vitoria Academic Center, Federal University of Pernambuco, Vitoria de Santo Antão 55608-680, Pernambuco, Brazil

**Keywords:** early-life brain injury, gut microbiota, gut–brain axis, maternal inflammation, neurodevelopment

## Abstract

Early-life brain injuries are major causes of long-term neurodevelopmental disorders such as cerebral palsy. Emerging evidence suggests these injuries can alter the gut microbiota composition, intestinal integrity, and neuroinflammatory responses. This systematic review evaluated the impact of early-life brain injuries on the gut microbiota in rodent models. A scientific literature search was conducted across Medline/PubMed, Web of Science, Scopus, and Embase. Initially, 7419 records were identified, and 21 eligible studies were included. Eligible studies focused on evaluating the microbiota alterations and related gut–brain axis markers at the neonatal or post-weaning stages. The data extraction and synthesis followed PRISMA guidelines. Most studies reported gut dysbiosis characterized by a decreased abundance of Bacteroidetes, and *Lactobacillus*. Alterations were associated with an increased gut permeability, reduced tight junction proteins, and elevated pro-inflammatory cytokines. Several studies showed reduced levels of short-chain fatty acids and metabolic pathway disruptions. Brain outcomes included neuroinflammation, white matter injury, altered gene expression, and impaired structural integrity. These results suggest that early-life brain injury induces complex alterations in the gut microbiota and its metabolic products, which may contribute to systemic and neuroinflammatory processes. Understanding these interactions offers insights into the pathophysiology of neurodevelopmental disorders and highlights the gut–brain axis as a potential target for early interventions.

## 1. Introduction

Early-life brain injury (ELBI) is the most recognized cause of mortality and severe, long-term neurological deficits in children, such as cerebral palsy (CP) [1,2]. ELBI results from a range of potential factors with complex mechanisms, primarily involving hypoxia-ischemia and inflammation [1], but also including toxic exposures and mechanical trauma [3,4], which can disrupt neurodevelopmental trajectories. In animal studies, these conditions are commonly replicated through experimental models such as hypoxia–ischemia, maternal immune activation (MIA), and traumatic brain injury (TBI) [5]. Furthermore, the developing brain is susceptible to intrinsic (genetic) and extrinsic (acquired) insults, which can have a lifelong impact on neurological function [6].

In recent years, increasing attention has been given to a bidirectional communication network connecting the central nervous system (CNS) and the gastrointestinal tract, the gut–brain axis (GBA) [7,8,9]. The resident microorganism communities inside the gut act as a key regulator of this axis [7], which encompasses bacteria, fungi, viruses, and other microorganisms [10]. These microbes influence brain function via neural, hormonal, and immune pathways [7,8], while the CNS, in turn, modulates gastrointestinal functions such as motility, secretion, and absorption [7].

The microbial colonization of the gut begins early, often before the maturation of neural systems [8]. It can influence brain development prenatally, via the maternal microbiota, and postnatally, through factors such as the delivery mode, breastfeeding, and antibiotic exposure [8]. These early-life exposures can rearrange the gut microbial composition and, consequently, impact neurodevelopmental trajectories [11,12]. The postnatal period represents a window of heightened plasticity in neuronal circuits, making the CNS especially sensitive to microbial and environmental influences [11].

The gut microbiota plays a key role in modulating immune responses, producing metabolites and neurotransmitters, and influencing the development of the brain [8,13]. It has been suggested that microbial metabolites help regulate the integrity of the blood–brain barrier (BBB) and affect paracellular permeability [13]. Furthermore, early microbial colonization appears to modulate neurogenesis in the hippocampus, a brain region critical for memory and learning [13]. The intestinal barrier also plays a crucial role in CNS homeostasis [7]. Dysbiosis, an imbalance in the gut microbial community, can lead to “leaky gut” syndrome, allowing the translocation of microbial components such as lipopolysaccharides (LPSs) into the bloodstream, which may trigger neuroinflammation [7,14]. Such inflammatory processes have been implicated in the pathophysiology of various neurodevelopmental and psychiatric disorders, including autism spectrum disorder (ASD) [7].

The developmental origins of the health and disease (DOHaD) hypothesis supports the notion that early-life exposures, including microbial, inflammatory, and environmental factors, can have long-lasting effects on health and neurodevelopment [15,16]. Promising studies suggest that the gut microbiome may actively regulate brain development, including cognitive functions and behavior [8,13]. Given these findings, we hypothesized that ELBIs, beyond their direct effects on the CNS, may also impact the structure and function of the gut microbiota, potentially influencing neurodevelopmental outcomes via the gut–brain axis. This review aimed to evaluate the impact of ELBI on the gut microbiota in rodent models. By analyzing taxonomic shifts, short-chain fatty acid (SCFA) profiles, and associated neurobiological changes, we seek to elucidate microbiota-mediated mechanisms linking peripheral and central responses to ELBI.

## 2. Materials and Methods

### 2.1. Systematic Review Report and Description of the Protocol

This systematic review follows the guidelines of the Preferred Reporting Items for Systematic Reviews and Meta-Analyses (PRISMA) statement to ensure transparency in reporting [17]. The review protocol was registered in the International Prospective Register of Systematic Reviews (PROSPERO) with the registration identification CRD420250610455.

### 2.2. Database Search Strategy

The scientific literature search was conducted in February 2025 across the following databases: Medline/PubMed, Web of Science, Scopus, and Embase. Appropriate search terms were applied to address the following research question: “How do early brain injuries influence gut microbiota, and what are the consequences of these changes on neurodevelopment?”. The search strategy incorporated MeSH (Medical Subject Headings), DeCS (Health Science Descriptors), and relevant keywords and synonyms tailored to each database (Table 1).

### 2.3. Eligibility

No restrictions were applied on language or publication year in the article selection process across databases. This process was conducted in two stages: (i) screening titles and abstracts; and (ii) full-text review of the studies. The entire eligibility process was performed by two independent reviewers (Souza and Silveira), with any inconsistencies resolved by a third reviewer (Pereira). Using the Rayyan platform (https://www.rayyan.ai/ accessed on 12 March 2025), the two primary reviewers made blinded decisions, and, only at the end, the third reviewer accessed the decisions to resolve potential conflicts.

In the first phase, the following inclusion criteria were applied: (i) experimental studies conducted on rodents; (ii) animals exposed to early brain injury (within the first 28 postnatal days); and (iii) studies investigating effects on microbiota. Studies identified in the search were excluded at this stage based on the following criteria: (i) studies involving humans; (ii) studies involving non-rodent animals; (iii) genetically modified animal species; (iv) non-original studies (e.g., reviews, letters to the editor, and opinion articles); (v) studies on unrelated diseases or conditions; (vi) animals without early brain injury; and (vii) studies not reporting microbiota outcomes (Table 2).

After the initial screening, studies were reviewed in full text, and only those fulfilling all inclusion criteria were retained: (i) the presence of control groups; (ii) a detailed description of the brain injury model; and (iii) outcomes related to microbiota. Studies presenting one or more exclusion criteria were removed: (i) genetically modified animal species; (ii) lack of information on the timing of brain injury; and (iii) no description of the tests used to assess microbiota.

### 2.4. Data Extraction

After selecting eligible articles, two independent reviewers (Souza and Silveira) used a specific spreadsheet (Microsoft Excel) to extract and organize relevant data, in the following order: (i) author’s last name and year of publication of the study; (ii) animals used (species, sex, and quantity of animals per experimental group); (iii) type of brain injury (model used, and age at which it occurred); (iv) experimental groups; (v) microbiota assessment methods; (vi) microbiota outcomes (biodiversity indicators and taxonomical composition. In addition to primary outcomes, secondary outcomes were also collected when reported: brain-related outcomes, microbial metabolite profiles (e.g., SCFAs), gut barrier integrity markers, intestinal cytokine levels, and systemic inflammatory markers. For the results and discussion, studies were grouped based on the developmental stage of the animals and the type of outcome assessed. When data was unclear or incomplete in the published articles, corresponding authors were contacted via email to request additional information or clarification.

### 2.5. Assessment of Methodological Quality

The methodological quality of the studies included in this review was evaluated by two researchers (Silveira and Pereira) using Syrcle’s Risk of Bias (RoB) tool, which consists of 10 assessment items. A “yes” response indicates a low risk of bias, a “no” response indicates a high risk of bias, and an “uncertain” response suggests insufficient information to determine the risk of bias [18]. The items assessed by this tool are as follows: (i) sequence generation; (ii) baseline and concealment characteristics; (iii) allocation concealment; (iv) accommodation for random allocation of animals; (v) blinding of caregivers and investigators involved in the research; (vi) randomized evaluation; (vii) blinding of the evaluator of the results; (viii) incomplete data results; (ix) selective communication of results; and (x) other sources of bias [18]. Due to the high heterogeneity among the included studies, especially in terms of microbiota assessment methods, it was not feasible to perform a meta-analysis. Therefore, the findings are organized by taxonomic levels and developmental stages.

## 3. Results

### 3.1. Identification and Study Selection

**Figure 1:**
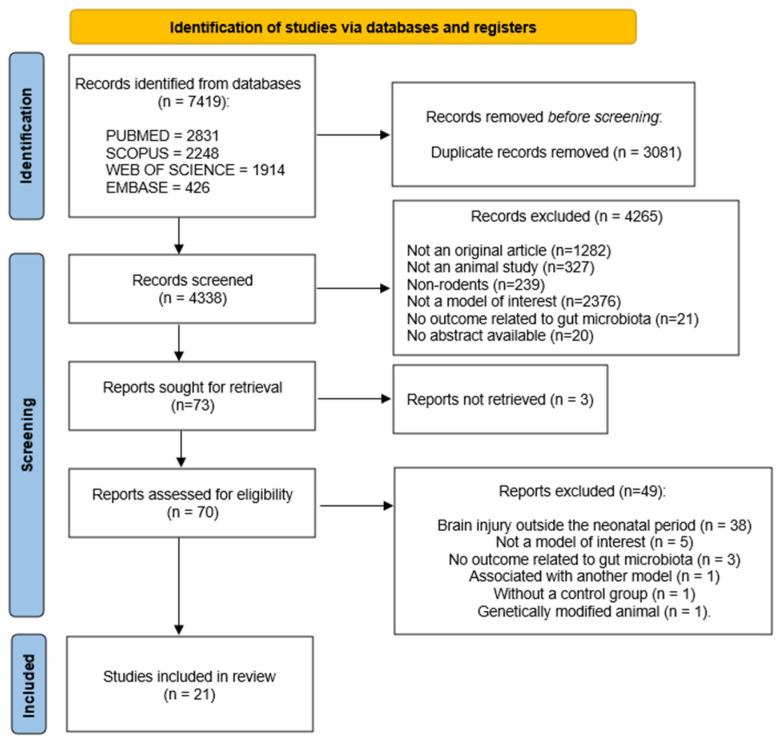
PRISMA flow diagram of the study identification and selection process via databases.

The process of study identification and selection is specified in the PRISMA flow diagram (Figure 1). A total of 7419 articles were identified in the four databases searched (Embase = 426; PubMed = 2831; Scopus = 2248; and Web of Science = 1914), with 3081 duplicates removed. The remaining 4338 articles were analyzed based on the eligibility criteria by analyzing the titles and abstracts. Of these, 70 articles were selected for a full-text assessment according to the predefined inclusion and exclusion criteria. After a detailed analysis, 49 articles also presented at least one exclusion criterion, resulting in a total of 21 studies included in this review (Figure 1).

### 3.2. Characteristics of Included Studies

A detailed and systematic summary of the main characteristics of the studies included in this review is presented in Table 3 and Supplementary Tables S1–S4. Among the 21 included studies, 10 used Sprague–Dawley rats [4,19,20,21,22,23,24,25,26,27], 7 used Wistar rats [28,29,30,31,32,33,34], 2 used C57BL6/J mice [35,36], 1 study used BALB/cByJ mice [37], and 1 study did not specified the animals’ species [3]. Regarding the sex of the animals, 11 studies included both male and female animals [19,20,21,23,25,26,28,31,32,35,36], 9 studies used only male rats [4,22,24,27,29,30,33,34,37], and 1 study did not specify [3].

### 3.3. Early-Life Brain Injury Models

Among the studies included, 11 employed models of neuroinflammation induced by exposure to LPS, valproic acid (VPA), or Poly I:C during gestation or the initial days of life [21,22,24,29,30,31,32,33,34,35,37], 6 studies used the hypoxia–ischemia model via unilateral carotid artery occlusion [20,23,26,28,36], 1 study used a spastic CP model [25], 1 used a chronic hypoxia model [27], 1 used a TBI model [4], and 1 used an neodymium exposure model [3].

### 3.4. Effect of Early-Life Brain Injury on Gut Microbiota (Primary Outcome)

#### 3.4.1. Shifts in Microbial Diversity/Richness (Alpha and Beta Diversity)

Alpha and beta diversity are widely used to quantify differences in microbiome composition, either within or between groups. Alpha diversity (within-sample) summarizes the structure of a microbial community in terms of richness (number of taxonomic groups), evenness (distribution of abundances of the groups), or both. The most commonly used alpha diversity metrics include Chao1, Simpson’s, and Shannon’s indices [38]. Differences in alpha diversity were estimated in 17 of the included studies, based on these three indices.

Eleven of these studies [4,19,20,21,23,26,29,30,32,34,37] reported no significant differences in alpha diversity between experimental and control groups. Huang et al. observed a transient increase in alpha diversity (Shannon index) at postnatal day 7 compared to day 3 within the Sham group, but not between experimental groups [21]. Some of these studies employed additional diversity indices beyond the three most used metrics (e.g., Observed species, Faith’s index, Pielou’s evenness, and Good’s coverage). A higher alpha diversity (Shannon, Simpson, Observed OTUs, and Chao indices) following experimental intervention was observed in only three studies [24,27,31], while a significant reduction (Shannon, Chao, Simpson, and Faith’s indices) was reported in three studies [3,25,33]. Additionally, Cuskelly et al. found that LPS-exposed females had an increase in alpha diversity compared to males (Shannon and Simpson indices) [31].

Four studies did not evaluate the alpha diversity; however, Li et al. reported a significant reduction in the total bacterial load in Poly I:C offspring, which may indirectly reflect a lower microbial richness [22]. Drobyshevsky et al., He et al., and Tartaglione et al. did not provide explicit alpha diversity analyses [28,35,36].

Beta diversity metrics (between-samples) summarize which samples differ from one another by considering sequence abundances or considering only the presence–absence of sequences. Commonly used beta metrics are the Bray–Curtis dissimilarity, Jaccard, unweighted UniFrac, and weighted UniFrac [38]. Of the 16 studies that reported beta diversity indices, 13 detected significant differences (e.g., UniFrac, PCoA, and NMDS) between the control and experimental groups [3,4,19,20,24,25,26,27,29,30,32,33,37] and 2 studies showed no significant difference in beta diversity [23,34]. Cuskelly et al. reported a significant difference in beta-diversity for females between the LPS and saline groups, and no change was observed in males [31]. In the remaining studies, beta diversity was not assessed [21,22,28,35,36].

#### 3.4.2. Differences in the Composition of Gut Microbial Taxa

- Phylum level

Fourteen studies reported significant differences in gut microbial composition at the phylum level. In the neonatal stage (up to postnatal day 28), increased abundances were observed for Proteobacteria [19,21,25,26], Bacteroidetes [21,24,35], Firmicutes [36], Fusobacteria [28], Actinobacteria [23], Campylobacterota, Desulfobacterota, and Elusimicrobiota [25]. In contrast, other studies reported a lower abundance of Bacteroidetes [19,23,26,36], Proteobacteria [36], Firmicutes [21], Chloroflexi [25], Verrucomicrobia, and Tenericutes [35].

In young and adult animals (from postnatal day 29 onward), higher abundances were reported for Firmicutes [23,37], Actinobacteria [23,37], Bacteroidetes [31], Fusobacteria [29], Campylobacterota [20], and Proteobacteria [23]. Conversely, lower abundances were observed for Actinobacteria [20,24,29,30], Bacteroidetes [23,37], Firmicutes [4,30], and Proteobacteria [30,31].

- Class level

Four studies reported significant differences in gut microbial composition at the class level. In the neonatal stage, He et al. found increased abundances for Fusobacteriia and Bacilli, and a decreased abundance of Clostridia [28].

In the young and adult stages, an increased abundance of Campylobacteria [20], Clostridia, and Coriobacteriia were reported [37]. In contrast, Coriobacteriia [20], Erysipelotrichia [24,37], and Bacteroidia [37] presented a lower abundance.

- Order level

Five studies reported significant differences in gut microbial composition at the order level. In the neonatal stage, increased abundances were observed for Lactobacillales [3,28], Clostridiales, Bacteroidales [3], Enterobacteriales, and Fusobacteriales [28]. Conversely, other studies reported a lower abundance of Clostridiales, Micromonosporales [28], Enterobacteriales, and Verrucomicrobiales [3].

In the young and adult stages, an increased abundance was observed for Clostridiales, Coriobacteriales [37], and Campylobacterales [20]. By contrast, Coriobacteriales [20], Erysipelotrichales [20,37], and Bacteroidales [37] presented a lower abundance.

- Family level

Fourteen studies reported significant differences in gut microbial composition at the family level. In the neonatal stage, increased abundances were observed for Enterobacteriaceae [19,26,28], Lactobacillaceae [26,28,35], Fusobacteriaceae [19,28], Helicobacteraceae [25], Prevotellaceae, Streptococcaceae, Vibrionaceae [19], Peptostreptococcaceae, Tannerellaceae [28], Lachnospiraceae, Oscillospiraceae, Desulfovibrionaceae, Elusimicrobiaceae, and Butyricicoccaceae [25], as well as Bacteroidaceae, Cyclobacteriaceae, Cytophagaceae, Lentimicrobiaceae, and Sphingobacteriaceae [35].

However, other studies reported reduced abundances of Prevotellaceae [25,27,28], Enterococcaceae [19,26], Clostridiaceae [25,35], Akkermansiaceae [19,35], Erysipelotrichaceae [24], Staphylococcaceae [25], Helicobacteraceae, Victivallaceae, Planococcaceae [19], Lachnospiraceae, Ruminococcaceae, Neisseriaceae, Micromonosporaceae [28], and other families such as Erwiniaceae, Kiloniellaceae, Mycoplasmataceae, and Rhizobiaceae [35].

In the young and adult stages, increased abundances were reported for Lactobacillaceae [4,23,33], Helicobacteraceae [20,23], Prevotellaceae, Ruminococcoceae [24], Fusobacteriaceae, Rikenellaceae [29], Bacteroidaceae, Bifidobacteriaceae [33], Spirochaetaceae, Veillonellaceae, Burkholderiaceae, Lachnospiraceae, Peptostreptococcaceae [23], Peptococcaceae, and Coriobacteriaceae [37].

Conversely, reduced abundances were reported for Bacteroidaceae [4,23], Prevotellaceae [23,37], Peptostreptococcaceae [23,24], Rikenellaceae [33,37], Corynebacteriaceae [29,33], Erysipelotrichaceae [20,29], Coriobacteriaceae [34], Micrococcaceae, Staphylococcaceae, Aerococcaceae [29], Atopobiaceae [20], Tannerellaceae, Muribaculaceae [23], Ruminococcaceae [37], and Mycoplasmataceae [35].

- Genus level

Fifteen studies reported significant differences in gut microbial composition at the genus level. In the neonatal stage, increased abundances were observed for *Lactobacillus* [3,28,32,35], *Bacteroides* [21,25,35], *Parabacteroides* [3,28,35], *Prevotella* [3,20], *Romboutsia* [28,32], *Blautia* [3,25], *Helicobacter* [25], *Ruminococcus* [35], *Fusobacterium*, *Escherichia_Shingella* [28], and *Alloprevotella* [27]. In contrast, other studies reported decreased abundances of *Bacteroides* [3,27], *Akkermansia* [3,35], *Lactobacillus* [21], *Rothia*, *Enterococcus*, *Escherichia_Shigella* [32], *Ruminococcus* [25], *Parabacteroides* [27], *Escherichia* [3], *Staphylococcus*, and *Prevotella* [25].

**Figure 2:**
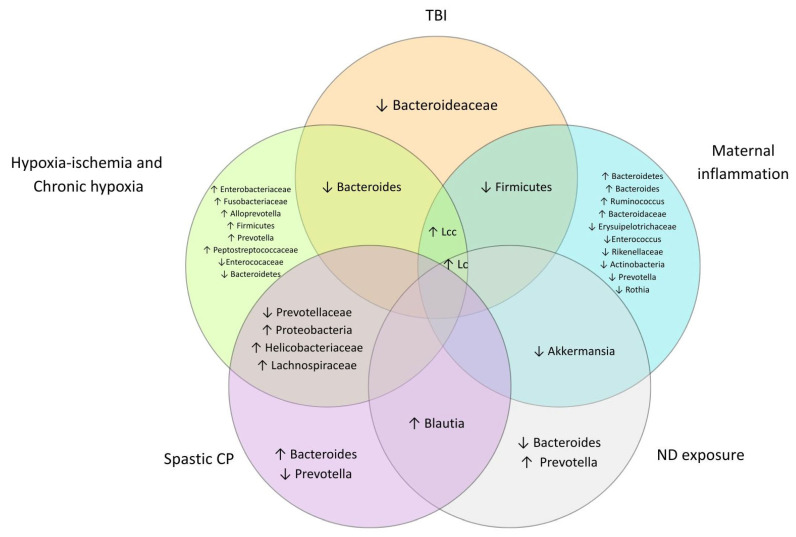
Main bacterial taxa reported across the included studies. Lcc refers to Lactobacillaceae and Lc refers to *Lactobacillus*. Arrows indicate the direction of relative abundance changes (↑ increased, ↓ decreased). Figure created in Biorender.com.

In the young and adult stages, studies reported increased abundances of *Lactobacillus* [4,22,33], *Ruminococcus* [37], *Alistipes* [29], *Helicobacter*, *Alloprevotella* [20], *Bacteroides*, and *Bifidobacterium* [22]. Conversely, other studies reported reduced abundances for *Bacteroides* [4,20,34], *Alloprevotella* [35], *Staphylococcus*, *Blautia* [29], *Alistipes* [20], and *Prevotella* [37]. Other genera showing significant differences in abundance are presented in Table 3. The main altered taxa identified across the experimental models are summarized in Figure 2, highlighting increases in Lactobacillaceae and *Lactobacillus*.

- Species level

Three studies reported significant differences in gut microbial composition at the species level, all of them conducted in the young and adult stages. Li et al. observed a significantly increased abundance of *Escherichia coli* [22]. Similarly, Zamudio-Flores et al. reported an increased abundance of *Prevotellaceae*_NK3B31_group_uncultured_bacterium [4]. Additionally, Romero-Miguel et al. reported an increased abundance of *Lactobacillus intestinalis*, along with a reduced abundance of *Corynebacterium stationis* [33].

### 3.5. Secondary Outcomes

To facilitate a clearer overview of the secondary outcomes reported across the included studies, these results have been compiled and summarized in Supplementary Table S5.

#### 3.5.1. Association Between Brain-Related Outcomes and Early-Life Brain Injuries

Several studies in this review investigated the impact of ELBI on brain structure, inflammation, and molecular signaling. These findings were grouped according to the developmental stage.

- Neonatal stage

Multiple studies demonstrated signs of neuroinflammation and structural damage following HI injury, MIA, LPS exposure, or toxic insults. Chen et al. reported an increased activation of microglia and astrocytes [19], while Lin et al. and Wei et al. reported elevated hippocampal levels of pro-inflammatory cytokines such as IL-6, TNF-α, LPS, and IL-1β [26,32]. Synaptic degeneration and neuronal loss were also observed in the hippocampus and cortex of HI animals [19,28].

Wei et al. further reported that HI injury induced a strong upregulation of Toll-like receptor pathways (TLR4, MyD88, and NF-κB), a marked loss of CA1 neurons, and an elevated number of Iba+ and TUNEL+ cells [26]. Gene expression analyses revealed enrichment in inflammatory and apoptotic signaling. Similarly, Tartaglione et al. and Yan et al. observed increased markers of inflammation and white matter injury (e.g., Iba1+, reduced MBP, and CC1+ cells), confirming demyelination and neuroinflammation [27,35]. Epigenetic disruptions were also detected, including altered histone crotonylation patterns [28]. Jia et al., in a neonatal model of neodymium exposure, found evidence of BBB dysfunction [3].

- Young and adult stages

Persistent neurobiological alterations were also observed beyond the neonatal period. Lee et al. demonstrated a reduced MBP+ area in the prefrontal cortex and thalamus of MIA offspring, indicating long-term hypomyelination [29,30]. Palanivelu et al., using diffusion tensor imaging, identified lower fractional anisotropy (FA) and a higher mean diffusivity (MD) in multiple brain regions, including the hippocampus and anterior cingulate cortex, along with sustained glial activation (GFAP+, Iba1+) [24]. These alterations were also found during the neonatal stage [24]. Prince et al. reported increased TMEM119 in the cerebellum and reduced Tph2+ cells in the raphe nuclei of VPA-exposed animals [37], suggesting a link between neuroinflammation and altered serotonin biosynthesis. Additionally, Romero-Miguel et al. found a reduced hippocampal volume [33], and Zamudio-Flores et al. described reduced CEA/cell-body area ratios in cortical and hippocampal neurons post-TBI [4].

#### 3.5.2. Association Between Intestinal Alterations and Early-Life Brain Injuries

- Neonatal stage:

Several studies reported structural and inflammatory changes in the intestines of animals exposed to early-life insults. In HI models, a histopathological analysis revealed increased tissue damage and higher histology scores in the colon [19,26], along with a reduced expression of tight junction proteins such as Occludin and ZO-1 [19,26]. Additionally, compromised intestinal barriers were observed through pathogenic LPS leakage into feces and serum [26].

In the LPS-induced neonatal inflammation model, intestinal disorganization, higher injury scores, and reduced ZO-1 expression were observed at early postnatal days [21]. Jia et al. reported a decreased number of goblet cells with thicker mucus layers in the colon [3]. Moreover, studies found elevated colonic levels of pro-inflammatory cytokines, including IL-6, IL-17a, IL-22, and TNF-α [19,32]. Drobyshevsky et al. also found a reduced villi density [36].

- Young and adult stages:

In later developmental stages, intestinal disturbances persisted or worsened. In animals exposed to Poly(I:C), the gut morphology was distorted, with severe cellular injury in the colon and increased mRNA and protein levels of IL-1β and TNF-α [22]. These animals also exhibited reduced levels of ZO-1 and claudins [22,26]. In the VPA model, it was observed that reduced ZO-1 protein levels in the ileum suggesting impaired tight junction integrity and increased intestinal permeability [37]. Additionally, Ni et al. reported inflammatory cell infiltration, and the upregulation of glycolytic and immune-related genes in the colon [23].

#### 3.5.3. Association Between Microbial Metabolites and Early-Life Brain Injuries

- Neonatal stage

Several studies demonstrated that ELBI induces substantial metabolic shifts in the gut microbiota during the neonatal period. One of the alterations involved a reduction in SCFAs. In neodymium-exposed neonates, Jia et al. observed decreased levels of acetic, propionic, butyric, and isobutyric acids, while the levels of valeric and isovaleric acid were elevated [3]. Similarly, Palanivelu et al. reported reduced levels of acetate and butyrate alongside elevated levels of formate at postnatal days 21, 35, and 49 [24], indicating a sustained impact on microbial metabolic function.

Beyond SCFAs, He et al. showed that HI injury significantly downregulated key microbial pathways, including butanoate metabolism, fatty acid biosynthesis, neuroactive ligand–receptor interaction, pantothenate and CoA biosynthesis, pentose phosphate pathway, and glycine, serine, and threonine metabolism, while HIF-1 signaling was upregulated [28]. Additionally, Lin et al. demonstrated that LPS exposure suppressed the production of 21 metabolites in the neonatal gut, although individual compounds were not specified [32].

In a model of chronic hypoxia, Yan et al. identified large-scale shifts in microbial metabolites using LC-MS, with 297 compounds increased and 610 decreased [27]. Notably, the bile acid cholic acid was significantly elevated in the brain, serum, and feces, and positively correlated with the abundance of *Bacteroides thetaiotaomicron* and *Parabacteroides distasonis*. These findings link early hypoxic injury to disruptions in bile acid metabolism, a key pathway involved in gut–brain communication and inflammation regulation.

Ni et al., using an HI model, reported a complex pattern of functional metabolic dysregulation [23]. There was an increase in microbial functions associated with susceptibility to bacterial infections. In addition, key pathways related to cellular processes and signaling were downregulated. Functions involved in the biosynthesis of antibiotics such as streptomycin, penicillin, cephalosporin, butirosin, and neomycin were also significantly reduced.

- Young and adult stages

In older animals, the metabolic consequences of ELBI remained evident. Ni et al. observed in HI-exposed animals an increase in functions relating to susceptibility to bacterial infections [23]. These changes were accompanied by reductions in functions related to cellular processes and signaling, and antibiotic biosynthesis, suggesting a dysbiotic shift toward a more inflammatory and less stable microbial environment.

Palanivelu et al. confirmed that SCFA findings persisted into the juvenile and early adult stages in ASD rats, pointing to a long-term metabolic alteration in the gut microbiota [24]. In turn, Prince et al. explored how these SCFA alterations correlated with specific taxa [37]. Positive associations were observed between propionic acid and *Prevotella*, butyric acid and Bacteroidales, and branched-chain SCFAs (e.g., isobutyric and isovaleric) and Peptococcaceae. Conversely, acetic acid was negatively associated with *Prevotella*, as well as *Ruminococcus* and Ruminococcaceae with butyrate and propionate, respectively.

#### 3.5.4. Association Between Systemic Inflammatory Markers and Early-Life Brain Injuries

- Neonatal stage

Several studies reported increased systemic inflammation following ELBI. Serum concentrations of inflammatory cytokines such as IL-1β, IL-6, TNF-α, and LPS were significantly elevated in injured animals [19,26,31,32]. Chu et al. also found an increase in IL-8 levels, and the IL-1β expression was higher at postnatal day 28 compared to day 14 [20]. Similarly, Tao et al. observed elevated IL-6, and TNF-α, as well as corticosterone (CORT) and adrenocorticotropic hormone (ACTH), suggesting hypothalamic–pituitary–adrenal (HPA) axis activation [25]. Palanivelu et al. reported elevated levels of IL-1β, IL-6, IFN-γ, and TNF-α in ASD animals, with progressive increases observed over time (until postnatal day 49) [24].

- Young and adult stages

In later developmental stages, systemic inflammatory alterations persisted. Palanivelu et al. observed the continued elevation of pro-inflammatory cytokines (IL-1β, IL-6, IFN-γ, and TNF-α) in ASD rats [24]. Interestingly, Cuskelly et al. observed a decrease in IL-6 in LPS-exposed males but not in females, suggesting a sexually dimorphic response in adulthood to neonatal LPS [31].

### 3.6. Risk of Bias Assessment

**Figure 3:**
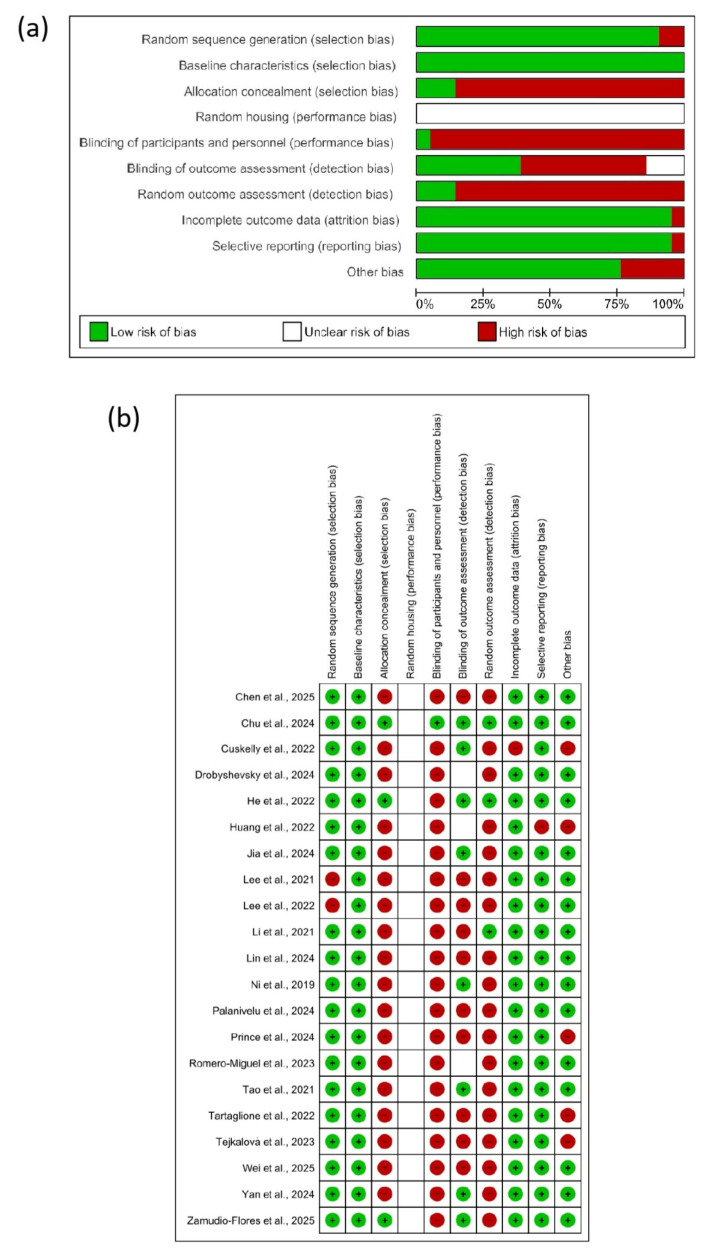
Risk of bias of included studies: percentage graph covering all included studies (**a**) and abstracts for individual studies (**b**) + (green) low risk of bias, (red) high risk of bias, and (no color) unclear risk of bias [3,4,19,20,21,22,23,24,25,26,27,28,29,30,31,32,33,34,35,36,37].

The risk of bias across the included studies is summarized in Figure 3, where Figure 3a presents a percentage ‘risk of bias’ plot covering all included studies and Figure 3b presents the ‘risk of bias’ summaries for individual studies. No studies were judged to be at a low risk of bias across all domains. According to selection bias, in random sequence generation, only two studies were considered at a high risk of bias (*n* = 2/21) [29,30], while the remaining studies described how sequence generation was performed (*n* = 19/21) [3,4,19,20,21,22,23,24,25,26,27,28,31,32,33,34,35,36,37]. All studies evaluated the baseline characteristics of the animals studied. Regarding allocation concealment, most studies were considered to be at a high risk of bias (*n* = 18/21) [3,19,21,22,23,24,25,26,27,29,30,31,32,33,34,35,36,37], while only three studies provided a clear description (*n* = 3/21) [4,20,28], presenting a low risk of bias. In detection bias, all studies presented an unclear risk of bias. Regarding the blinding of the participants and personnel, only one study addressed it perfectly, presenting a low risk of bias (*n* = 1/21) [20], while the remaining studies exhibited a high risk of bias (*n* = 20/21). Detection bias was variable across studies, with 10 articles that did not perform blinding of the outcome assessment (*n* = 10/21) being considered to have a high risk of bias [19,22,24,26,29,30,32,34,35,37], or three studies being considered to have an unclear risk of bias (*n* = 3/21) [21,33,36] or eight studies included in the study being considered to have a low risk of bias (*n* = 8/21) [3,4,20,23,25,27,28,31]. More than half of the included studies did not highlight the blinding of the random outcome assessment (*n* = 18/21) [3,4,19,21,23,24,25,26,27,29,30,31,32,33,34,35,36,37], while only three studies showed a low risk of bias (*n* = 3/21) [20,22,28]. In terms of attrition bias, only one study did not adequately present the results with clarity of the data, thus being considered to be at a high risk of bias (*n* = 1/21) [31], while the other studies properly displayed incomplete results. Among all the studies included, in terms of reporting bias, only one study exhibited a high risk of bias for not making the article available for free (*n* = 1/21) [21]; however, the other studies properly displayed the free manuscript. Another bias was identified among five studies, in which there was a conflict of interest between the authors and financial institutions, indicating a high risk of bias (*n* = 5/21) [21,31,34,35,37]; however, in the remaining studies, we did not observe any other bias. Most studies demonstrate a satisfactory conciseness and symmetry, consistent with a low publication bias.

## 4. Discussion

This review aimed to evaluate the impact of ELBI on gut microbiota composition in rodent models. Considering the growing evidence linking gut dysbiosis to neurodevelopmental outcomes via the gut–brain axis, we systematically analyzed preclinical studies to identify the taxonomic and functional microbial changes following ELBI, as well as the associated alterations in intestinal and neural outcomes. By synthesizing these findings, this review seeks to advance the understanding of the microbiota-mediated mechanisms underlying brain injury in early life.

We found that ELBI consistently altered the gut microbiota diversity and composition in rodent models. While the alpha diversity remained unchanged in most studies, beta diversity differences were reported across nearly all experiments. This suggests that ELBI does not necessarily reduce microbial richness but induces broad taxonomic shifts and a high interindividual variability, a hallmark of dysbiosis [39,40]. Nonetheless, reductions in alpha diversity were observed in specific models, such as those involving Poly I:C, neodymium exposure, and spastic CP, suggesting that the severity, timing, or duration of injury may modulate microbial richness. Although a low alpha diversity is commonly linked to poor host outcomes, its role as a biomarker for neurodevelopmental disorders remains inconclusive [40].

At the taxonomic levels, the results varied across developmental stages and models. Firmicutes and Bacteroidetes are the most typical phyla in the healthy gut microbiota, followed by Actinobacteria, Proteobacteria, and Fusobacteria [41,42]. Here, Firmicutes showed contradictory results: three studies reported an increased abundance [23,36,37], while another three reported decreases [4,21,30]. Higher levels of Firmicutes have also been observed in patients with mild cognitive impairment [43], linked to the pathogenesis of neurodegenerative diseases [44], potentially through the modulation of neuroactive metabolite production and the modification of the host neurotransmitter circuitry [45,46].

Despite four studies reporting a higher abundance of the phylum Bacteroidetes, a lower abundance was described in five studies [4,19,23,36,37]. Ni et al. observed the reduction at both developmental stages [23]. Bacteroidetes are Gram-negative bacteria that exhibit pro-inflammatory properties due to endotoxins and influence cytokine production [47]. These divergent results may be due to differences in the experimental models, injury timing, or host age at assessment. Methodological variability across studies, including sequencing and analysis techniques, may also contribute to these inconsistencies.

From an immunological perspective, Bacteroidetes participate in immunomodulatory processes. Their surface components, including LPS, can activate host cell receptors, enhance immune responses via cytokine synthesis [47], and induce an inflammatory response locally and at distant sites [48]. Notably, the four studies that reported increased Bacteroidetes employed inflammation-based models [21,24,31,35]. Given that Gram-negative bacteria often thrive in pro-inflammatory environments, this may partly explain the observed elevation in this phylum abundance.

The reduction in Bacteroidetes may be particularly relevant, given their important functional roles in neurodevelopmental and immune modulation. Bacteroidetes are known producers of SCFAs such as acetate and propionate, which have been shown to protect neurons from oxidative stress [9,49]. Moreover, Bacteroidetes influences the expression of neuroplasticity-related genes such as BDNF, syntaxin, and drebrin in the hippocampus [50], indicating that the microbial modulation may affect behavior and cognitive performance [51]. Reductions in this phylum have been associated with impaired structural and functional plasticity of the hippocampus [9,52].

In the neonatal stage, an increase in Proteobacteria [19,21,23,25,26], a phylum associated with structural dysbiosis and intestinal inflammation [53,54], was frequently observed. At the family level, frequent increases were noted in Enterobacteriaceae (phylum Proteobacteria) [19,26,28] and Lactobacillaceae (phylum Firmicutes) [4,23,26,28,35]. Enterobacteriaceae are among the most overgrown symbionts in many conditions involving inflammation, and the inflamed gut appears to provide a favorable environment for the expansion of Enterobacteriaceae [42]. Its expansion has been related to the disruption of intestinal mucosa tight junctions, increased intestinal permeability [55], and elevated LPS production [26], exacerbating intestinal inflammation [55]. Moreover, increases in Proteobacteria and Enterobacteriaceae have been reported in gut inflammation and functional disorders such as irritable bowel syndrome, and are implicated in the pathophysiology of these disorders [19,56].

Lactobacillaceae is a family of lactic acid bacteria that naturally inhabit the intestinal microbiota of humans and many animals [57]. These bacteria are widely recognized for their beneficial effects on gut health, including the modulation of immune responses, protection against pathogenic microorganisms, and preservation of the intestinal barrier [9,57]. Notably, Lactobacillaceae also contributes to the production of butyrate, a SCFA with anti-inflammatory properties that plays a key role in maintaining epithelial integrity and mucosal immunity [9].

In addition to their intestinal functions, Lactobacillaceae may also influence the central nervous system. Studies suggest that their presence can modulate the expression of neurotrophic factors such as BDNF and proBDNF [9], which are crucial for synaptic plasticity, learning, and memory. Therefore, both Bacteroidetes and Lactobacillaceae may act as beneficial microbial taxa with potential roles in supporting brain development and neuroplasticity through microbiota–gut–brain axis signaling [9].

Interestingly, the genus *Lactobacillus* (family Lactobacillaceae) was also frequently increased across all stages [3,4,22,27,28,33,35]. *Lactobacillus* species contribute to the maintenance of the microbial balance by competing with pathogens, enhancing mucus secretion [58], causing anti-inflammatory properties [59], reinforcing the intestinal barrier [58,60], and producing anti-inflammatory metabolites such as SCFAs [61]. Some strains, such as *L. gasseri*, also reduce microglial activation and promote BDNF expression [60,62]. The increase in *Lactobacillus* observed may reflect a compensatory response in trying to avoid gut and brain inflammation. However, certain *Lactobacillus* strains have been implicated in excessive immune activation or opportunistic infections, especially in immunocompromised individuals [63,64]. Recent reviews suggest that CNS injury can lead to systemic immune suppression, while unresolved neuroinflammation persists in perinatal brain injury [65,66], creating a paradoxical immune environment where microbial taxa such as *Lactobacillus* may act differently. In this context, their proliferation could reflect a compensatory anti-inflammatory adaptation or disrupted host–microbe signaling.

These taxonomic alterations of Proteobacteria, Enterobacteriaceae, Lactobacillaceae, and *Lactobacillus* coincide with evidence of damage to the intestinal barrier, a critical interface in gut–brain communication [13]. In some of the studies reviewed, there was consistent reporting of gut barrier impairment, including structural and morphological disorganization [3,21,22], disrupted villi [21,36], and mucosal inflammation [3]. Molecular analyses revealed a decreased expression of tight junction proteins, such as ZO-1 [19,21,22,37], Occludin [19,26], and Claudins [22,26], and increased levels of pro-inflammatory cytokines, such as IL-6 [32], IL-17α, IL-22 [19], TNF-α [22,32], and IL-1β [22]. These findings indicate an environment of sustained immune activation and compromised intestinal integrity. Interestingly, Jia et al. reported an increased thickness of the colonic mucus layer [3]. This alteration may reflect another compensatory response to epithelial damage and inflammation. Despite this, the same study also showed a reduced number of goblet cells, suggesting a potential imbalance in mucus production. Together with the other findings, these results reinforce the hypothesis of a dysfunctional intestinal barrier in ELBI models. Additionally, both the decreased integrity of the intestinal barrier and increased intestinal inflammation may be associated with increased serum levels of pro-inflammatory mediators [19].

Here, systemic inflammation was observed following ELBI, in the neonatal and later developmental stages. Studies reported elevated serum levels of pro-inflammatory cytokines, including IL-1β, IL-6, TNF-α, and IFN-γ, as well as circulating LPS [19,24,25,26,32]. These findings suggest that increased intestinal permeability may facilitate the entry of pro-inflammatory mediators into the brain via the bloodstream, thereby promoting systemic inflammation and participating in mediating neural damage [19]. The expansion of Enterobacteriaceae, observed in some studies included in this review, may help to explain this response, as this family is known to promote inflammatory responses in many diseases, through their secretion of metabolites or immune modulation capabilities [19,26]. Tao et al., using a spastic CP model, also found concomitant elevations in CORT and ACTH, suggesting the upregulation of the HPA axis [25]. Notably, hyperactive HPA axis activation has been linked to depression-like behaviors and altered stress responses [25,67]. The persistence of these alterations into the juvenile and adult stages [35] indicates the presence of a chronic inflammatory state that may act as a mediator between intestinal dysbiosis and systemic inflammation and neuroinflammatory outcomes.

Moreover, studies reported a decreased abundance of Prevotellaceae in both the neonatal and young and adult stages [23,25,27,28,37]. This family is known for its SCFA-producing capacity [68] and important roles in intestinal barrier integrity and intestinal homeostasis, regulating the immune system, and reducing inflammation [20,69,70]. Moreover, the reduction in Prevotellaceae and increase in Lactobacilliaceae are related to neuroinflammation and were discovered in neurodegenerative diseases such as Parkinson’s disease [71,72]. Nevertheless, the decreased abundance of this family could represent a link between gut dysbiosis and neuroinflammation.

Interestingly, *Prevotella* (Prevotellaceae family) is considered a signature taxon of CP [20], and it was found to be increased in only two studies included in this review [3,20], both in neonatal stages. These studies used models based on hypoxia–ischemia and maternal exposure to neodymium, respectively. In contrast, two other studies reported a decrease in *Prevotella*: Tao et al., who used a surgical model of spastic CP involving motor cortex ablation [25], and Prince et al., who employed prenatal exposure to VPA [37]. These discrepancies may reflect the model-specific effects, where *Prevotella* expansion is promoted by systemic or metabolic insults, but suppressed in models involving direct cortical damage or other factors, such as the developmental stage, particularly since Prince et al. examined the microbiota in juvenile rats [37]. *Prevotella* has been implicated in the modulation of inflammatory pathways and may be involved in the pathophysiology of mental disorders through mechanisms linked to the gut–brain axis [73,74]. Previous research identified an increased abundance of *Prevotella* in the gut microbiota of children with CP [75], suggesting a potential link between its persistence and sustained dysbiosis. This prolonged imbalance may contribute to the enhanced permeability of the gut–brain barrier, thereby exacerbating neuroimmune disturbances [73]. Further research is needed to determine whether *Prevotella* shifts functionally between protective and pathogenic roles on the host context and environmental factors.

In addition to those findings, two studies in our review reported decreased levels of SCFAs [3,24], such as acetate, butyrate, and propionate, which are known for reducing the inflammatory response, promoting CNS plasticity, increasing the hematoencephalic permeability, enhancing epithelial integrity, increasing mucus production, and regulating intestinal motility [3,72]. These reductions were accompanied by increased formate, and valeric and isovaleric acids [3,24]. Functional pathway analyses revealed the downregulation of microbial metabolic functions. In HI-exposed animals, two studies found the reduced expression of pathways involved in butanoate metabolism, fatty acid synthesis [28], and functions related to cellular processes and signaling [23], and an increase in functions relating to susceptibility to bacterial infections [23]. These findings suggest that ELBI alters the gut microbiota’s taxonomic composition and disrupts its functional activity, shifting its role from a homeostatic symbiont to a potential disruptor. For instance, Prince et al. demonstrated significant correlations between altered SCFA levels and specific microbial taxa [37], such as the negative association between acetic acid and *Prevotella*, which may indicate a reduction in the microbiota’s capacity to produce metabolites essential for maintaining intestinal and neural homeostasis.

Moreover, the bile acid metabolism appeared disrupted. Yan et al. found that chronic hypoxia led to the accumulation of cholic acid, a primary bile acid with a crucial role in fetal brain development and inflammation [27], in the brain, serum, and feces. This was associated with a reduced abundance of *Bacteroides thetaiotaomicron* and *Parabacteroides distasonis*, species involved in bile acid conversion via 7α-dehydrogenase. As a result, the decreased enzymatic activity led to primary bile acid accumulation, which has been linked to neurodevelopmental disorders [27].

Consistent with these peripheral alterations described, ELBI models showed neuroinflammatory and neuropathologic changes, which may reflect the consequences of gut–brain axis disruption. The increased expression of pro-inflammatory cytokines (e.g., IL-6, and TNF-α), toll-like receptors, and downstream mediators were observed in hippocampal and cortical tissue [26,32,36]. Several studies reported microgliosis and astrogliosis, marked by elevated Iba1+, GFAP+, CD68+, TMEM119+, and TREM2+ cells [19,24,35].

Structural degeneration was also evident. Histological and imaging data revealed synaptic alterations, reduced neuronal density, demyelination, and increased cell death [19,27,28,29,30], alongside the downregulation of tight junction proteins (Occludin, ZO-1) in brain tissue, suggesting a BBB compromise [3,26]. Furthermore, reductions in hippocampal volume and altered DTI metrics reinforced the presence of macrostructural alterations in the brain [24,33].

At the molecular level, several studies reported epigenetic dysregulation (e.g., increased H3K9cr and decreased H3K18cr) and the downregulation of neurotrophic and plasticity-related genes, suggesting long-lasting impairments in neuronal growth and connectivity [28,32,35]. Notably, sex-specific and regional differences were also described. For instance, Tartaglione et al. found heightened glial activation markers in the female hippocampus and cerebellum during the early stages, followed by attenuated responses later in life [35].

Taken together, these findings suggest that ELBI triggers a complex cascade of gut–brain axis disruption, involving microbial imbalance, barrier dysfunction, altered metabolite profiles, and persistent neuroinflammation. These alterations might underline the behavioral and cognitive deficits observed in both experimental models and clinical populations affected by early neurodevelopmental insults. However, while associations between ELBI and gut dysbiosis are evident, the precise mechanisms by which brain injury influences the intestinal microbiota remain poorly elucidated. It is possible that neuroinflammation, altered neuroendocrine signaling, and systemic immune changes contribute to reshaping the microbial environment, but further mechanistic studies are required to clarify these pathways.

## 5. Limitations

Our systematic review has limitations that must be acknowledged. First, there was substantial heterogeneity among the included studies in terms of animal models, experimental protocols, analytical methods for the microbiota assessment, and developmental stages assessed. These differences limited the possibility of direct comparisons or the meta-analytical synthesis of the findings.

Second, in the study by Tao et al., although statistical differences in the microbial composition were reported, the authors did not explicitly indicate in which experimental groups each bacterial taxon was significantly altered [25]. This lack of detailed group-wise reporting reduces the interpretability and reproducibility of the findings and limits the inclusion of those results in our comparative synthesis.

A third limitation was the handling of treatment groups in certain studies. In Jia et al., three distinct doses of neodymium were used to induce early-life exposure, creating three experimental groups [3]. However, during the microbiota analysis, all neodymium-exposed animals were aggregated into a single group. This approach may have masked potential dose-dependent effects on microbial composition and limited the granularity of the findings.

A further limitation involved the study by Cuskelly et al., in which some microbiota-related results could be extracted; however, several relevant findings remained inaccessible due to the blurred supplementary figures and the lack of complete descriptions in the main text [31]. We contacted the corresponding author to request clarification, but no response had been received by the time of this manuscript submission.

Lastly, while most studies focused on bacterial taxonomic shifts, relatively few explored functional consequences such as microbial metabolites, host–microbiota signaling pathways, or the role of non-bacterial microorganisms (e.g., viruses and fungi), which could provide additional mechanistic insights.

## 6. Conclusions

This systematic review highlights the complex and multifactorial impact of ELBI on gut microbiota composition, intestinal barrier integrity, microbial metabolism, and systemic and neuroinflammatory responses in rodent models. Although the alpha diversity changes were inconsistent, nearly all studies reported significant alterations in beta diversity and specific taxonomic shifts, particularly involving Bacteroidetes, Proteobacteria, Enterobacteriaceae, Prevotellaceae, Lactobacillaceae, and *Lactobacillus*. These microbial changes were frequently associated with epithelial barrier disruption, reduced levels of beneficial metabolites such as SCFAs, and increased systemic and central inflammation.

The findings also suggest that ELBI-induced gut dysbiosis may act as both a consequence and a modulator of neurodevelopmental impairment, mediated by mechanisms involving systemic immune activation, HPA axis dysregulation, and persistent neuroinflammation. Notably, the observed increase in *Lactobacillus* brings questions about the role of commensal bacteria in injury contexts, potentially functioning as anti-inflammatory agents or opportunistic contributors to dysbiosis, depending on the immune environment.

Together, these data highlight the gut–brain axis as a critical interface in the pathophysiology of perinatal brain injury and suggest that microbial markers and metabolites could represent novel targets for early diagnosis and intervention. Future research should explore the causal relationships within this axis and evaluate the therapeutic potential of microbiota modulation in the prevention or attenuation of neurodevelopmental sequelae.

## Figures and Tables

**Table 1:** Search strategy.

Component	Terms/Boolean Operators
Early Brain Injury	Early Brain Injury OR Traumatic Brain Injury OR Traumatic Encephalopathy OR Cerebral Palsy OR Brain Hypoxia Ischemia OR Brain Hypoxia-Ischemia OR Cerebral Ischemia-Hypoxia OR Cerebral Ischemia Hypoxia OR Neonatal Asphyxia OR Perinatal Asphyxia OR Lipopolysaccharide Maternal Exposure OR Neuroinflammatory OR LPS Exposure OR Neuroinflammation
AND Microbiota	Microbiota OR Microbial Community OR Microbiome OR Microbial Community Structure OR Gastrointestinal Microbial Community OR Gut Microbiome OR Gastrointestinal Microbiota OR Gut Microbiota OR Intestinal Microbiome OR Intestinal Microbiota OR Gut Dysbiosis OR Gut-Brain Axis OR Intestinal Dysbiosis
AND Animals	animal experimentation OR Animals OR animal population groups OR rat OR rats OR animal OR animals OR mice OR mouse

Note: the search terms were adjusted as needed to align with the specific requirements of each database.

**Table 2:** Inclusion and exclusion criteria.

Category	Inclusion	Exclusion
Population	- Rodent animals	- Non-rodent animals - Genetically modified animals - Humans
Interventions	- Brain lesion in the perinatal period	- Animals subjected to more than one experimental model - Neurodegenerative models - Other diseases
Quality of studies	- Presence of an appropriate control group	- Studies without a control group - Insufficient methodological detail for critical appraisal
Outcomes	- Changes in intestinal microbiota - Neuroinflammation and/or neurodevelopment	- Studies that do not mention these outcomes
Study type	- Original studies	- Non-original studies (e.g., reviews, letters to the editor, and opinion articles)

This table summarizes the eligibility criteria used to identify the studies included in this systematic review.

**Table 3:** Characteristics of the included studies evaluating gut microbiota in ELBI models.

Study Characteristics	Model Description	Experimental Groups	Microbiota Assessment Methods	Microbiota Outcomes	
				Biodiversity Indicators	Taxonomical Composition
**Chen et al., 2025** [19] - **Sprague**–Dawley rats - Both sexes - HI = 8 animals - Sham = 8 animals Total = 16 animals	Hypoxia–ischemia: At P7, rats were subjected to the Rice–Vannucci modeling method to create an animal model of HIBD. Sham rats underwent anesthesia, and their left carotid artery was exposed but not ligated.	- HI = hypoxia–ischemia group - Sham = sham control group	16S rRNA gene sequencing (feces collected at P10) V3–V4 regions of 16S rRNA genes Seq: Illumina MiSeq Pipeline: FASTP, UPARSE v9.2.64, SILVA v138.1, and OmicShare tools	Alpha diversity - Chao1, Shannon and Simpson indices. - No significant change between the groupsBeta diversity - Bray–Curtis and ANOSIM. - The microbiota composition is different between the groups).	HI vs. Sham (*p* < 0.05) - ↑ HI: p_Proteobacteria, f_Fusobacteriaceae, f_Enterobacteriaceae, f_Prevotellaceae, f_Streptococcaceae, and f_Vibrionaceae. - ↓ HI: f_Akkermansiaceae, f_Enterococcaceae, f_Victivallaceae, f_Helicobacteraceae, and f_Planococcaceae.
**Chu et al., 2024** [20] **- Sprague–Dawley rats** - Both sexes - CP = 6 animals - Con = 6 animals Total = 12 animals	Hypoxia–ischemia: CP was modeled in neonatal rats (P7) by ligating the left carotid artery. After anesthesia, the artery was isolated and ligated, and the wound was closed. The rats were then rewarmed and placed in a hypoxia chamber (92% nitrogen, and 8% oxygen) for 1 h.	- CP = cerebral palsy group - Con = control group	16S rRNA gene sequencing (feces collected at P100, P107, P114, and P121) V4 region of bacterial 16S rRNA, 18S V5 region of eukaryotic 18S rRNA and ITS1 and ITS2 (fungal diversity) Seq: Illumina NovaSeq 6000 Pipeline: FASTP, USEARCH v10, and UPARSE v7.1	Alpha diversity - Chao1, Shannon, and Simpson indices. - No significant change in alpha diversity between the control and the PC group.Beta diversity - UniFrac, PCoA, and NMDS. - Both groups showed a distinct gut microbiota composition, although some similarities were observed.	CP vs. Con (*p* < 0.05) - ↑ CP: p_Campilobacterota, f_Helicobacteraceae, c_Campylobacteria, *g_Helicobacter*, o_campylobacterales, *g_*GCA_900066575, *g_Roseburia*, *g_Lachnospiraceae*_NK4A136_group, g_(Eubacterium)_xylanophilum_group, *g_Prevotella*, *g_Treponema*, *g_Prevotellaceae*_NK3B31_group, *g_Desulfovibrio*, and *g_Alloprevotella* (the last 4 were absent in the control group). - ↓ CP: f_Erysipelotrichaceae, o_Erysipelotrichales, *g_Dubosiella*, o_Coriobacteriales, c_Coriobacteriia, p_Actinobacteriota, *g_Enterorhabdus*, *g_Coriobacteriaceae*_UCG_002, f_Atopobiaceae, *g_Clostridia*_UCG-014, *g_Alistipes*, and *g_Bacteroides*.
**Cuskelly et al., 2022** [31] - Wistar rats - Both sexes - LPS-fem = 8 animals - LPS-male = 8 animals - Sal-fem = 8 animals - Sal-male = 8 animals Total = 32 animals	LPS exposure: On P3 and P5, pups were separated from their dams and placed in an incubator at 34 °C to maintain body temperature. Pups were administered an intraperitoneal injection of LPS (*Salmonella enterica*, serotype enteritidis, dissolved in sterile pyrogen-free saline) at 0.05 mg/kg in 0.02 mL, or an equivolume of 0.9% saline.	- LPS-fem = lipopolysaccharide female group - LPS-male = lipopolysaccharide male group - Sal-fem = saline female group - Sal-male = saline male group	16S rRNA gene sequencing (feces collected at P90) V6–V8 regions Seq: Illumina V3 MiSeq Pipeline: QIIME2, and DADA2	Alpha diversity - Chao1, Shannon, and Simpson - Increased richness and evenness of LPS groups by Shannon and Simpson indices (*p* < 0.05). Beta diversity - PCoA plot of Bray–Curtis - Significant difference for LPS-fem compared to Sal-fem, but no difference was observed in the males’ groups.	- Firmicutes was the most abundant phylum, followed by Bacteroidetes and Proteobacteria. LPS-fem vs. Sal-fem (*p* < 0.05) - p_Bacteroidetes (↑ LPS-fem) and p_Proteobacteria (↓ LPS-fem).
**Drobyshevsky et al., 2024** [36] **- C57BL/6J mice** - Both sexes - Sham = 6 animals - HI = 31 animals Total = 37 animals	Hypoxia–ischemia: At P10, the left carotid artery was permanently ligated with a double knot silk suture 7–0 under isoflurane anesthesia. Lidocaine was added to the wound for local analgesia. The wound was sutured, and pups returned to dam for recovery and nursing for 3 h, followed by 60 min of hypoxia with 8% O_2_ at 37 °C.	- Sham = sham group (no brain injury) - HI = hypoxia–ischemia group	16S rRNA gene sequencing (feces collected at P13) Regions, sequencing platform, and pipeline not specified	Not assessed	HI vs. Sham (*p* < 0.05) - p_Firmicutes (↑HI) and p_Proteobacteria (↓ HI)
**He et al., 2022** [28] - Wistar rats - Both sexes - HI = 50 animals - Sham = 30 animals Total = 80 animals	Hypoxia–ischemia: At P7~P10, pups were anesthetized, and the left carotid artery was permanently ligated. After suturing the wound, the pups were returned to their mother. Once awake, they were placed in a hypoxia chamber (8% O_2_) for 2 h. The Sham group had artery exposure but without ligation or hypoxia.	- Sham = sham control group - HI = hypoxic–ischemic group	16S rRNA gene sequencing (feces collected from colon and rectum at P21~P24). V3–V4 regions of bacterial 16S rRNA gene Seq: Illumina MiSeq PE300 Pipeline: QIIME 1.8.0, VSEARCH, RDP Classifier, and Greengenes database	Not assessed	HI vs. Sham (*p* < 0.05) - ↑ HI: p_Fusobacteria, c_Fusobacteriia, c_Bacilli, o_Enterobacteriales, o_Lactobacillales, o_Fusobacteriales, f_Enterobacteriaceae, f_Lactobacillaceae, f_Tannerellaceae, f_Fusobacteriaceae, f_Peptostreptococcaceae, *g_Parabacteroides*, *g_Lactobacillus*, *g_Fusobacterium*, *g_Romboutsia*, *g_Escherichia_Shingella*, *g_Burkholderia_Caballeronia_Paraburkholderia*, and *g_Holdemanella*. - ↓ HI: c_Clostridia, o_Clostridiales, o_Micromonosporales, f_Prevotellaceae, f_Lachnospiraceae, f_Ruminococcaceae, f_Neisseriaceae, f_Micromonosporaceae, *g_Pygmaiobacter*, *g_Peptococcus*, *g_Anaerotruncus*, *g_Harryflintia*, *g_Acetatifactor*, *g_Butyricicoccus*, *g_Neisseria*, *g_Ruminiclostridum*_6, *g_Ruminiclostridum*_9, *g_Oscillibacter*, *g_Micromonospora*, *g_Oscillospira*, *g_Ruminococcus*_1, *g_Anaerostipes*, and *g_Prevotella*_9.
**Huang et al., 2022** [21] **- Sprague–Dawley rats** - Both sexes - LPS = not specified - Sham = not specified Total = 136 animals	LPS exposure: At gestation day 15, pregnancy rats were randomly assigned to two groups, receiving a single 700 μg/kg intraperitoneal injection of LPS or the equivalent volume of saline for sham controls, respectively.	- LPS = lipopolysaccharide group - Sham = sham control group	16S rRNA gene sequencing (feces collected at P3 and P7) V3–V4 regions of bacterial 16S rRNA gene Seq: Illumina MiSeq Pipeline: FLASH, Trimmomatic, USEARCH v7.0, RDP Classifier, SILVA v128, and Mothur v1.30.1	Alpha diversity - Shannon index. - Sham at P7 was higher than at P3 (*p* < 0.05). - No significant change in alpha diversity between the groups at different ages. Beta diversity Not assessed	- The phyla of Firmicutes, and Proteobacteria were the most abundant in the two groups. LPS vs. Sham (*p* < 0.05) - ↑ LPS P3: p_Bacteroidetes, and *g_Bacteroides* - ↓ LPS P3: *g_Actinomyces*, and *g_Enterococcus*. - ↑ LPS P7: p_Bacteroidetes, p_Proteobacteria, *g_Bacteroides*, and *g_Escherichia-Shigella*. - ↓ LPS P7: p_Firmicutes, *g_Lactobacillus*, *g_Rodentibacter*, and *g_Veillonella*.
**Jia et al., 2024** [3] - Species not specified - ND (0.5, 1 and 2%) = not specified - Con = not specified Total = not specified	Nd_2_O_3_ exposure: The administration of Nd_2_O_3_ occurred during the gestation and lactation periods (22 days +21 days), with dosing frequencies of 0, 50, 100, and 200 mg/(kg·d). Distilled water of equal volume was administered to the control group.	- ND = neodymium group - Con = control group	16S rRNA gene sequencing (feces collected at P21) Regions not specified Seq: not specified Pipeline: QIIME2, classify-sklearn, and Greengenes	Alpha diversity - Chao1, Observed species, Shannon, Simpson, Faith’s PD, Pielou’s evenness, and Good’s coverage - Only Faith’s PD index showed a statistical difference between the groups. Beta diversity - PCoA. - Differences between the two groups of samples.	- The phyla of Firmicutes and Bacteroidetes were the most abundant in the two groups. ND vs. Con (*p* < 0.05) - ↑ ND: o_Clostridiales, o_Lactobacillales, o_Bacteroidales, *g_Lactobacillus*, *g_Prevotella*, *g_Parabacteroides*, and *g_Blautia*. - ↓ ND: o_Verrucomicrobiales, o_Enterobacteriales, *g_Bacteroides*, *g_Escherichia*, and *g_Akkermansia*.
**Lee et al., 2021** [29] **- Wistar rats** - Male rats - MIA = 5 animals - Con = 5 animals Total = 10 animals	LPS exposure: On gestational day 9.5, 500 μg/kg LPS (Escherichia coli O127:B8) or PBS was injected intraperitoneally into pregnant rats.	- MIA = maternal inflammation activation group - Con = control group	16S rRNA Gene Sequencing and Next-Generation Sequencing (feces collected at 7-week-old *) V3–V4 regions of bacterial 16S rRNA genes Seq: Illumina MiSeq system Pipeline: Cutadapt, DADA2, SILVA v128, DECIPHER, phangorn, phyloseq, GUniFrac, and vegan	Alpha diversity - Chao1, Shannon, Simpson, and Observed indices. - No significant change in alpha diversity between the groups. Beta diversity - Unweighted and weighted UniFrac and PCoA. - The fecal microbiota profile of MIA group was different from Con group.	MIA vs. Con (*p* < 0.05) - ↑ LPS: p_Fusobacteria, f_Fusobacteriaceae, f_Rikenellaceae, *g_Ruminococcus*_1, *g_Fusobacterium*, *g_Acetatifactor*, *g_Alistipes*, and *g*_DNF00809. - ↓ LPS: p_Actinobacteria, f_Micrococcaceae, f_Staphylococcaceae, f_Aerococcaceae, f_Corynebacteriaceae, f_Erysipelotrichaceae, *g_Coprococcus*_3, *g_Rothia*, *g_Sellimonas*, *g_Staphylococcus*, *g_Aerococcus*, *g_Corynebacterium*_1, *g_Candidatus_Stoquefichus*, and *g_Blautia*.
**Lee et al., 2022** [30] **- Wistar rats** - Male rats - MIA = 5 animals - Con = 5 animals Total = 10 animals	LPS exposure: 500 μg/kg LPS (from *Escherichia coli* O127:B8; Sigma) or PBS was injected intraperitoneally into the pregnant rats on gestation day 9.5.	- MIA = maternal inflammation activation group - Con = control group	16S rRNA gene sequencing and Next-Generation Sequencing (feces collected at 7-week-old) V3–V4 regions of bacterial 16S rRNA genes Seq: Illumina MiSeq Pipeline: Cutadapt, DADA2, SILVA v138, ECIPHER, phangorn, phyloseq, GUniFrac, and vegan	Alpha diversity - Chao1, Shannon, and Simpson indices. - No significant change in alpha diversity between the groups. Beta diversity - NMDS with Bray–Curtis. - The fecal microbiome profile of MIA rats was different from the controls.	MIA vs. Con (*p* < 0.05) - ↓ LPS: p_Firmicutes, p_Proteobacteria, p_Actinobacteriota, f_Enterobacterales, f_Erysipelotrichaceae, and o_Lactobacillales.
**Li et al., 2021** [22] - Sprague–Dawley rats - Male rats - PolyIC = 10 animals - Con = 10 animals Total = 20 animals	Poly I:C exposure: The rat dams were intravenously injected with 10 mg/kg poly I:C (Sigma–Aldrich) in saline, or an equal amount of saline solution according to previous study.	- PolyIC = Poly I:C group - Con = control group	DNA extraction and analysis (feces collected between P59 and P60) 16S region not performed Seq: not applicable Pipeline: RT-qPCR (SYBR Green), and species-specific primers	Not assessed	PolyIC vs. Con (*p* < 0.05) - ↑ PolyIC: *g_Escherichia. coli*, *g_Lactobacillus* spp., *g_Bifidobacterium spp*., and *g_Bacteroides spp*. - The total number of bacteria was reduced in MIA offspring (*p* = 0.0163).
**Lin et al., 2024** [32] - Wistar rats - Both sexes - LPS = 6 animals - Con = 6 animals Total = 12 animals	LPS exposure: On P5 and P6, intraperitoneal injections of LPS (L2630, Sigma–Aldrich, St. Louis, MO, USA) dissolved in 0.9% NaCl was administered to neonatal rats at a dose of 200 ng/g body weight per day. Rats in the control group were administered intraperitoneal injections of 0.9% NaCl.	- LPS = lipopolysaccharide group - Con = control group	16S rRNA gene sequencing and bioinformatics (colonic content collected at P7) V3–V4 regions of bacterial 16S rRNA Seq: Illumina MiSeq Pipeline: not specified	Alpha diversity - Richness, Shannon, Simpson, Pielou, Invsimpson, Chao1, ACE, and goods_coverage - No significant change between the groups. Beta diversity - PCA showed distinct differences between the groups.	LPS vs. Con (*p* < 0.05) - ↑ LPS: *g_Romboutsia*, *g_Clostridium_sensu_stricto*_1, and *g_Lactobacillus*. - ↓ LPS: *g_Rothia*, *g_Escherichia-Shigella*, and *g_Enterococcus.*
**Ni et al., 2019** [23] **- Sprague–Dawley rats** - Both sexes - HI = 10 animals - Con = 10 animals Total = 20 animals	Hypoxia–ischemia: On P14, rats underwent left carotid artery ligation under anesthesia, followed by 3 h of recovery. They were then placed in a hypoxia chamber (8% O_2_) for 2 h at 37 °C. Sham groups had an incision without artery manipulation.	- HI = hypoxia–ischemia group- - Con = control–sham group	- DNA extraction and qPCR analysis of the feces (collected at P16, P17, P18, P23, P29, and P36) and cecal and colonic contents V3–V4 regions prokaryotic and bacterial 16S rRNA Seq: Illumina MiSeq Pipeline: QIIME2 and DADA2	Alpha diversity - Shannon, Simpson, Evenness and Faith’s indices. - No significant change between the groups. Beta diversity - Weighted Unifrac. - No significant changes between the groups.	HI vs. Con (*p* < 0.05) Fecal content: - p_Bacteroidetes (↓HI on P16, P23. and P29) and p_Actinobacteria (↑HI on P21). Colonic and cecal contents: - ↑ HI: p_Firmicutes, p_Actinobacteria, p_Proteobacteria, f_Peptostreptococcaceae, f_Spirochaetaceae, f_Lactobacillaceae, f_Veillonellaceae, f_Burkholderiaceae, f_Lachnospiraceae, and f_Helicobacteraceae. - ↓ HI: f_Tannerellaceae, f_Muribaculaceae, f_Bacteroidaceae, and f_Prevotellaceae.
**Palanivelu et al., 2024** [24] - Sprague–Dawley rats - Male rats - ASD = 15 animals - Con = 15 animals Total = 30 animals	VPA exposure: Pregnant rats received a single intraperitoneal injection of sodium valproic acid (Depakene) (NaVPA, Merck, Darmstadt, Germany) at a dosage of 500 mg kg^−1^ body weight. NaVPA was dissolved in 0.9% physiological saline to achieve a concentration of 150 mg/mL, with a pH of 7.3, and was administered on gestational days 12–13.	- ASD = autism model group - Con = control group	16S rRNA gene sequencing and Next-Generation Sequencing (feces collected at P21, P35, and P49). V3–V4 region of bacterial 16S rRNA gene Seq: Illumina MiSeq Pipeline: cutadapt, DADA2, SILVA v132, DECIPHER, RAxML, phyloseq, GUniFrac, LEfSe, and GraPhlAn	Alpha diversity - Observed OTUs and Chao1: increased in species richness in ASD rats at P21, P35, and P49. - Shannon and Simpson: higher diversity in ASD rats at all time points (*p* < 0.01). Beta diversity -PCoA and UniFrac: differences in microbiome composition between the groups at P35 and P49.	- Bacteroidetes and Firmicutes were the predominant phyla in both groups from P21 to P49. - p_Firmicutes predominated at P21 and p_Bacteroidetes became dominant on P35 and P49. ASD vs. Con (*p* < 0.05) - ↑ ASD P21: p_Bacteroidetes. - ↓ ASD P21: *g_Erysipelotrichaceae*. - ↑ ASD P35: p_Tenericutes, f_Prevotellaceae, and f_Ruminococcoceae. - ↓ ASD P35: p_Actinobacteria, c_Erysipelotrichia, f_Peptostreptococcoceae, and f_Christensenellaceae. - ↑ ASD P49: f_Prevotellaceae and *g_Erysipelotrichia*. - ↓ ASD P49: f_Lachnospiraceae.
**Prince et al., 2024** [37] - BALB/cByJ mice - Male rats - VPA = 6 animals - PBS Control = 3 animals Total = 9 animals	VPA exposure: On G11, dams were injected subcutaneously with 600 mg/kg VPA to induce an autistic-like phenotype in the offspring or PBS as a control.	- VPA = valproic acid group - PBS = phosphate-buffered saline group	16S rRNA gene sequencing (cecal samples collected at P50) Regions not specified Seq: not specified Pipeline: QIIME 1.8	Alpha diversity - Shannon index and Reciprocal Simpson index. - No significant change in alpha diversity between the groups. Beta diversity - Bray–Curtis and PCoA. - The microbial community structure between samples showed clustering differences between groups (*p* < 0.001).	VPA vs. PBS (*p* < 0.05) - ↑ VPA: p_Firmicutes, p_Actinobacteria, c_Clostridia, c_Coriobacteriia, o_Clostridiales, o_Coriobacteriales, f_Peptococcaceae, f_Coriobacteriaceae, *g_Ruminococcus*, and *g_Adlercreutzia*. - ↓ VPA: p_Bacteroidetes. c_Bacterioidia, c_Erysipelotrichi, o_Erysipelotrichales, o_Bacteroidales, f_Prevotellaceae, f_Rikenellaceae, f_Ruminococcaceae, and *g_Prevotella.*
**Romero-Miguel et al., 2023** [33] - Wistar rats - Male rats - MIS = 6~12 animals - Con = 6~12 animals Total = 12~24 animals	Poly I:C exposure: On gestational day 15 (GD15), Poly I:C (4 mg/kg, Sigma–Aldrich, Madrid, Spain) or saline solution was administered i.v. to pregnant Wistar rats.	- MIS = Maternal Immune Stimulation group - Con = control group	16S rRNA Sequencing for Metagenomics (the collected between P110–120) Regions not specified Seq: Ion Torrent PGM Pipeline: not specified	Alpha diversity - Shannon index and Chao index The bacterial richness was reduced in MIS rats compared to Con (*p* < 0.05). Beta diversity - PCA of the Bray–Curtis Distance: - MIS animals presented the highest data dispersion, indicating differences in the gut microbiota composition associated with the maternal immune challenge.	- Bacteroidetes and Firmicutes populations were the most abundant phyla in all groups, followed by Proteobacteria and Actinobacteria. MIS vs. Con (*p* < 0.05) - ↑ MIS: f_Bacteroidaceae, f_Bifidobacteriaceae, f_Lactobacillaceae, f_Thermoanaerobacteraceae, *g_Lactobacillus*, and *L. intestinalis*. - ↓ MIS: f_Rikenellaceae, f_Corynebacteriaceae, f_Clostridiales XVI, f_Veillonellaceae, *g_Acetatifactor*, and *Corynebacterium stationis*.
**Tao et al., 2021** [25] - Sprague–Dawley rats - Both sexes - CP = ~12 animals - Con = 6 animals Total = ~18 animals	**Spastic CP: Rats were anesthetized, fixed on a device, and a 2 cm skull incision was made to expose the motor cortex. A hole was drilled, the cortex was removed, and the site was managed with saline and closed with a gelatin sponge.**	**- CP = cerebral palsy group** **- Con = control group**	**16S rRNA gene sequencing (feces collected at the P26).** **V3–V4 region of bacterial 16S rRNA gene** **Seq: Illumina MiSeq** **Pipeline: Trimmomatic, FLASH, UPARSE v7.1, and Majorbio Cloud**	**Alpha diversity** **- Chao index and Simpson index: increased in the Simpson index in control group when compared to CP group (*p* < 0.05).** **Beta diversity** **- PCA: No significant differences between the groups.** **- PLS-DA: The two groups could be distinguished and clustered into two groups.**	**CP vs. Con (*p* < 0.05)** **- ↑ CP: p_Campylobacterota, p_Proteobacteria, p_Desulfobacterota, p_Elusimicrobiota, f_Lachnospiraceae, f_Oscillospiraceae, f_Helicobacteraceae, f_Desulfovibrionaceae, f_Elusimicrobiaceae, f_Butyricicoccaceae, *g_Bacteroides*, *g_Blautia*, *g_Helicobacter*, and *g_Orea*.** **- ↓ CP: p_Chloroflexi, f_Prevotellaceae, f_Staphylococcaceae, f_Clostridiaceae, *g_Prevotella*, *g_Ruminococcus*, and *g_Staphylococcus*.**
**Tartaglione et al., 2022** [35] - C57BL6/J mice - Both sexes - Poly IC = 12 animals - Con = 12 animals Total = 24 animals	Poly I:C exposure: At gestational day 12.5 pregnant mice received a single injection of Poly I:C [(potassium salt; Sigma–Aldrich, #P9582), (20 mg/kg, i.p.)] or vehicle (0.9% NaCl).	- Poly IC = polyinosinic:polycytidylic group - Con = control group	16S rRNA sequencing and analysis (feces were collected on P28 and P120) V3–V4 region of bacterial 16S rRNA Seq: Illumina MiSeq Pipeline: FastQC, BBDuk, BWA-MEM, and GAIA 2.0	Not assessed	- Firmicutes and Bacteroidetes were the two dominant phyla in both groups at P28 and P120. - The Bacteroidetes/Firmicutes ratio was higher in PolyI:C mice at P28. - At P120, most of the differences observed between Poly I:C and Con mice at P28 were no longer detectable. Poly IC vs. Con (*p* < 0.05) - ↑ PolyIC P28: p_Bacteroidetes, f_Bacteroidaceae, f_Cyclobacteriaceae, f_Cytophagaceae, f_Lactobacillaceae, f_Lentimicrobiaceae, f_Sphingobacteriaceae, *g_Bacteroides*, *g_Lactobacillus*, *g_Nitritalea*, *g_Paludibacter*, *g_Parabacteroides*, *g_Ruminococcus*, *g_Sporocytophaga*, *g_Turicibacter*, *g_Desulfotomaculum* (females), *g_Fretibacter* (males), and *g_Lentimicrobium* (females). - ↓ PolyIC P28: p_Verrucomicrobia, p_Tenericutes, f_Akkermansiaceae, f_Clostridiaceae, f_Erwiniaceae, f_Kiloniellaceae, f_Mycoplasmataceae, f_Rhizobiaceae, *g_Akkermansia*, *g_Anaerofilum*, *g_Anaerotruncus*, *g_Anaerocolumna*, *g_Butyricicoccus*, *g_Clostridium*, *g_Faecalicatena*, *g_Fodinicurvata*, *g_Flintibacter*, *g_Phocea*, *g_Intestinibacillus*, *g_Mycoplasma*, *g_Tyzzerella*, *g_Ureaplasma*, and *g_Liberibacter* (females). - ↓ PolyIC P120: f_Mycoplasmataceae, *g_Ureaplasma*, *g_Mycoplasma*, *g_Alloprevotella*, and *g_Parasutterella*
**Tejkalová et al., 2023** [34] - Wistar rats - Male rats - LPS = 35 animals - Con = 35 animals Total = 70 animals	LPS exposure: LPS was dissolved in 0.9% NaCl and administered intraperitoneally (i.p.) at a dose of 2 mg/day/kg body weight (b.w.) to neonatal rats for 5 consecutive days (P5–9).	- LPS = lipopolysaccharide group - Con = control group	16S rRNA gene sequencing (feces were collected on P60 and P70) V4–V5 regions of bacterial 16 S rRNA Seq: Ion Torrent PGM Pipeline: DADA2, QIIME2 v2020.2, VSEARCH, and Greengenes v13_8	Alpha diversity - Shannon index, ASV, and Faith’s index of phylogenetic diversity. - No significant differences were found between the groups. Beta diversity - Jaccard’s nonphylogenetic distance matrix and PCoA. - No significant differences were found between the groups.	- Firmicutes were detected as the dominant phylum in all groups. - p_Firmicutes and p_Bacteroidetes (especially o_Clostridiales and o_Bacteroidales) were predominant in all groups. LPS vs. Con (*p* < 0.05) - ↑ LPS P70: *g_Oscillospira* - ↓ LPS P70: o_Actinomycetales, f_Coriobacteriaceae, and *g_Bacteroides.* - No differences were found between the LPS and Con groups in P60.
**Wei et al., 2025** [26] **- Sprague–Dawley rats** - Both sexes - HI group = 6 animals - Sham = 6 animals Total = 12 animals	Hypoxia–ischemia: The left common carotid artery was ligated in rats on postnatal day 7 under 3% isoflurane anesthesia. Following a 1 h recovery period with their dams, the rats were subsequently placed in a hypoxic chamber with 8% oxygen and maintained at 37 °C for 2 h.	- Sham = sham operation group - HI = hypoxia–ischemia group	16S rRNA sequencing using fecal samples collected at P10 V3–V4 region of bacterial 16S rRNA genes Seq: Illumina MiSeq Pipeline: not specified	Alpha diversity - Shannon and Simpson index - There was no difference between the HI and Sham groups. Beta diversity - PCoA. - Segregation in the microbial community structures between the groups (*p* = 0.002).	HI vs. Sham (*p* < 0.05) - ↑ HI: p_Proteobacteria, f_Enterobacteriaceae, and f_Lactobacillaceae. - ↓ HI: p_Bacteroidetes and f_Enterococcaceae.
**Yan et al., 2024** [27] **- Sprague–Dawley rats** - Male rats - NG = 10 animals - HG = 10 animals Total = 20 animals	Chronic hypoxia: Newborn male rats were housed in a hypoxic chamber under 10.5% O_2_ or in ambient air for 24 days, corresponding to the human neonatal stage.	- NG = normoxia group - HG = hypoxia group	16S rRNA gene sequencing using fecal samples collected at P24 V3–V4 or V4–V5 regions of bacterial 16S rRNA genes Seq: not specified Pipeline: Trimmomatic, FLASH, and QIIME1.8	Alpha diversity - Chao1, Simpson, and Shannon indices. - Shannon and Simpson indices were altered. Beta diversity - ANOSIM and PCoA. - Separation and clear microbial composition differences between the groups.	- Bacteroidetes/Firmicutes ratio in the hypoxic group rats was decreased (*p* < 0.01). HG vs. NG (*p* < 0.05) - ↑ HG: *g_Alloprevotella*, and *g_Prevotella*-1. - ↓ HG: f_Prevotellaceae, *g_Bacteroides*, and *g_Parabacteroides*.
**Zamudio-Flores et al., 2025** [4] - Sprague–Dawley rats - Male rats - TBI = 6 animals - Sham = 6 animals Total = 12 animals	TBI: The rats were anesthetized with isoflurane (5% induction, and 2% maintenance) and placed in a stereotaxic frame. A craniectomy was performed over the right hemisphere. A moderate TBI was induced using a Leica Impact One™ device (5 mm flat tip, 2.5 mm depth, 4 m/s velocity). The body temperature was maintained at 37 ± 0.5 °C. Sham animals underwent identical procedures without impact.	- TBI = traumatic brain injury group - Sham = sham control group	16S rRNA gene sequencing (fecal samples collected from the caecum on P42) V1–V4 regions of bacterial 16S rRNA fragments Seq: Illumina MiSeq Pipeline: DADA2 (QIIME2), VSEARCH, and SILVA v138	Alpha diversity - Shannon and Simpson index. - There was no difference between the TBI and Sham groups. Beta diversity - Bray–Curtis NMDS. - TBI group was different from Sham group (*p* = 0.04).	TBI vs. Sham (*p* < 0.05) - ↑ TBI: f_Lactobacillaceae, *g_Lactobacillus*, and *s_Prevotellaceae*_NK3B31_group_uncultured_bacterium. - ↓ TBI: p_Firmicutes, f_Bacteroidaceae, and *g_Bacteroides*.

* Age was estimated based on a similar protocol described by Lee et al., 2022 [30], due to the absence of this information in Lee et al., 2021 [29]. Bolded author names indicate the primary citation of each included study. Underline highlights (1) the alpha and beta diversity sections, and (2) the experimental model used in each study. ↑ increased, ↓ decreased.

## Data Availability

No new data were created or analyzed in this study.

## Supplementary Materials

The following supporting information can be downloaded at: https://www.mdpi.com/article/10.3390/cells14141063/s1. Table S1. PubMed Search Strategy. Table S2. Scopus Search Strategy. Table S3. Web of Science Search Strategy. Table S4. EMBASE Search Strategy. Table S5. Summary of secondary outcomes reported in the included studies.

## Abbreviations

The following abbreviations are used in this manuscript:

ACADS	acyl-CoA dehydrogenase short chain
ACC	anterior cingulate cortex
ACTH	adrenocorticotropic hormone
ANOSIM	analysis of similarities
ASD	autism spectrum disorder
ASV	amplicon sequence variant
BBB	blood–brain barrier
BDNF	brain-derived neurotrophic factor
CA1	Cornu ammonis 1 (hippocampus)
CD68	Cluster of differentiation 68
CNS	central nervous system
CORT	corticosterone
CP	cerebral palsy
DeCS	Health Science Descriptors
DEGs	differentially expressed genes
DOHaD	developmental origins of health and disease
DTI	Diffusion Tensor Imaging
ELBI	Early-life brain injury
FA	Fractional anisotropy
GBA	Gut–brain axis
GFAP	Glial fibrillary acidic protein
GSEA	gene set enrichment analysis
H3K9cr	Histone H3 lysine 9 crotonylation
H3K18cr	Histone H3 lysine 18 crotonylation
HI	Hypoxia–ischemia
HPA	Hypothalamic–pituitary–adrenal
Iba+	Ionized calcium-binding adaptor molecule
IFN-γ	Interferon gama
IL	Interleukin
KEGG	Kyoto Encyclopedia of Genes and Genomes
LC-MS	Liquid chromatography–mass spectrometry
LPS	Lipopolysaccharide
MBP	Myelin basic protein
MD	mean diffusivity
MeSH	Medical Subject Headings
MIA	Maternal immune activation
MyD88	Myeloid differentiation primary response 88
ND	Neodimium
NF-κB	Nuclear factor kappa-light-chain-enhancer of activated B cells
NMDS	Nonmetric multidimensional scaling
OTU	Operational Taxonomic Unit
PCA	Principal component analysis
PCoA	Principal coordinates analysis
PLS-DA	Partial Least Squares Discriminant Analysis
PolyIC	polyinosinic:polycytidylic acid
PRISMA	Preferred Reporting Items for Systematic Reviews and Meta-Analyses
qPCR	Quantitative Polymerase Chain Reaction
SCFA	short-chain fatty acids
Seq	sequencing plataform
TBI	traumatic brain injury
Th	T helper
TLR	Tool-like receptors
TMEM119	Transmembrane protein 119
TNF-α	tumor necrosis factor alpha
Tph2+	Tryptophan hydroxylase 2
TREM2	Triggering Receptor Expressed on Myeloid cells 2
TUNEL	Terminal Deoxynucleotidyl Transferase-Mediated dUTP Nick End Labeling
VPA	valproic acid
ZO-1	Zonula occludens-1

## References

[1] Berger I., Peleg O., Ofek-Shlomai N. (2012). Infammation and early brain injury in term and preterm infants. Isr. Med. Assoc. J..

[2] Tataranno M.L., Vijlbrief D.C., Dudink J., Benders M.J.N.L. (2021). Precision Medicine in Neonates: A Tailored Approach to Neonatal Brain Injury. Front. Pediatr..

[3] Jia Y., Cao J., Guo Y., Wu L., Du X., Tang B., Xia B., Deng Y. (2024). Intergenerational crosstalk of brain-gut axis in parental Nd_2_O_3_ exposure-induced offspring neurotoxicity and cognitive dysfunction: A mechanistic study. Front. Public Health.

[4] Zamudio-Flores J., Cerqueda D., Phillips-Farfán B., Guerrero-Flores S., Salinas-García A.F., Meléndez-Herrera E., Sélem-Mojica N., Kline A.E., Lajud N. (2025). Environmental enrichment-induced cognitive recovery after a moderate pediatric traumatic brain injury is associated with the gut microbiota and neuroinflammation. Exp. Neurol..

[5] Santos da Silva Calado C.M., Manhães-de-Castro R., Souza V.D.S., Cavalcanti Bezerra Gouveia H.J., da Conceição Pereira S., da Silva M.M., de Albuquerque G.L., Lima B.M.P., de Lira A.V.S.M., Toscano A.E. (2025). Early-life malnutrition role in memory, emotional behavior and motor impairments in early brain lesions with potential for neurodevelopmental disorders: A systematic review with meta-analysis. Nutr. Neurosci..

[6] Russ J.B., Ostrem B.E.L. (2023). Acquired Brain Injuries Across the Perinatal Spectrum: Pathophysiology and Emerging Therapies. Pediatr. Neurol..

[7] Wang Q., Yang Q., Liu X. (2023). La microbiota, el eje cerebro intestino y los trastornos del neurodesarrollo. Protein Cell.

[8] Mallick R., Basak S., Das R.K., Banerjee A., Paul S., Pathak S., Duttaroy A.K. (2024). Roles of the gut microbiota in human neurodevelopment and adult brain disorders. Front. Neurosci..

[9] Putri S.S.F., Irfannuddin I., Murti K., Kesuma Y., Darmawan H., Koibuchi N. (2023). The role of gut microbiota on cognitive development in rodents: A meta-analysis. J. Physiol. Sci..

[10] Davenport E.R., Sanders J.G., Song S.J., Amato K.R., Clark A.G., Knight R. (2017). The human microbiome in evolution. BMC Biol..

[11] Damiani F., Cornuti S., Tognini P. (2023). The gut-brain connection: Exploring the influence of the gut microbiota on neuroplasticity and neurodevelopmental disorders. Neuropharmacology.

[12] Tognini P. (2017). Gut microbiota: A potential regulator of neurodevelopment. Front. Cell. Neurosci..

[13] Iliodromiti Z., Triantafyllou A.R., Tsaousi M., Pouliakis A., Petropoulou C., Sokou R., Volaki P., Boutsikou T., Iacovidou N. (2023). Gut Microbiome and Neurodevelopmental Disorders: A Link Yet to Be Disclosed. Microorganisms.

[14] Fasano A. (2020). All disease begins in the (leaky) gut: Role of zonulin-mediated gut permeability in the pathogenesis of some chronic inflammatory diseases. F1000Research.

[15] Oldenburg K.S., O’Shea T.M., Fry R.C. (2020). Genetic and Epigenetic Factors and Early Life Inflammation as Predictors of Neurodevelopmental Outcomes. Semin. Fetal Neonatal Med..

[16] dos Santos Júnior J.P., dos Santos Júnior O.H., Silva-Araujo E.R., Cavalcanti Bezerra Gouveia H.J., Lacerda D.C., Visco D.B., Pontes Silva P.B., Cadena-Burbano E.V., Amaral de Souza Gonzaga Paz I.A., de Souza S.L. (2025). Phenotypic plasticity: Historical context, theories and DOHaD. Brain Res..

[17] Rethlefsen M.L., Kirtley S., Waffenschmidt S., Ayala A.P., Moher D., Page M.J., Koffel J.B., Blunt H., Brigham T., Chang S. (2021). PRISMA-S: An extension to the PRISMA Statement for Reporting Literature Searches in Systematic Reviews. Syst. Rev..

[18] Hooijmans C.R., Rovers M.M., de Vries R.B.M., Leenaars M., Ritskes-Hoitinga M., Langendam M.W. (2014). SYRCLE’s risk of bias tool for animal studies. BMC Med. Res. Methodol..

[19] Chen A., Teng C., Wei J., Wu X., Zhang H., Chen P., Cai D., Qian H., Zhu H., Zheng X. (2025). Gut microbial dysbiosis exacerbates long-term cognitive impairments by promoting intestinal dysfunction and neuroinflammation following neonatal hypoxia-ischemia. Gut Microbes.

[20] Chu C., Huang S., Wang X., Zhao G., Hao W., Zhong Y., Ma Z., Huang C., Peng Y., Wei F. (2024). Randomized controlled trial comparing the impacts of *Saccharomyces boulardii* and *Lactobacillus rhamnosus* OF44 on intestinal flora in cerebral palsy rats: Insights into inflammation biomarkers and depression-like behaviors. Transl. Pediatr..

[21] Huang Q., Lu S., Zhu Y., Wei B., Chen Y., Bai F. (2022). Bacterial endotoxin-induced maternal inflammation leads to fetal intestinal injury and affects microbial colonization in the neonatal period. J. Matern. Neonatal Med..

[22] Li W., Chen M., Feng X., Song M., Shao M., Yang Y., Zhang L., Liu Q., Lv L., Su X. (2021). Maternal immune activation alters adult behavior, intestinal integrity, gut microbiota and the gut inflammation. Brain Behav..

[23] Ni Y., Wang Z., Ma L., Yang L., Wu T., Fu Z. (2019). Pilose antler polypeptides ameliorate inflammation and oxidative stress and improves gut microbiota in hypoxic-ischemic injured rats. Nutr. Res..

[24] Palanivelu L., Chen Y.Y., Chang C.J., Liang Y.W., Tseng H.Y., Li S.J., Chang C.W., Lo Y.C. (2024). Investigating brain–gut microbiota dynamics and inflammatory processes in an autistic-like rat model using MRI biomarkers during childhood and adolescence. Neuroimage.

[25] Tao D., Zhong T., Pang W., Li X. (2021). Saccharomyces boulardii improves the behaviour and emotions of spastic cerebral palsy rats through the gut-brain axis pathway. BMC Neurosci..

[26] Wei J., Chen A., Huang D., Teng C., Cai D., Wu X., Wang T., Hu W., Huang Z., Wang P. (2025). Gut microbiome-derived lipopolysaccharides aggravate cognitive impairment via TLR4-mediated inflammatory signaling in neonatal rats following hypoxic-ischemic brain damage. Brain. Behav. Immun..

[27] Yan Y., Zheng X., Liu G., Shi G., Li C., Chen H., He X., Lin K., Deng Z., Zhang H. (2024). Gut microbiota-derived cholic acid mediates neonatal brain immaturity and white matter injury under chronic hypoxia. iScience.

[28] He X., Zhang T., Zeng Y., Pei P., Liu Y., Jia W., Zhao H., Bi M., Wang S. (2022). Sodium butyrate mediates histone crotonylation and alleviated neonatal rats hypoxic–ischemic brain injury through gut–brain axis. Front. Microbiol..

[29] Lee G.A., Lin Y.K., Lai J.H., Lo Y.C., Yang Y.C.S.H., Ye S.Y., Lee C.J., Wang C.C., Chiang Y.H., Tseng S.H. (2021). Maternal immune activation causes social behavior deficits and hypomyelination in male rat offspring with an autism-like microbiota profile. Brain Sci..

[30] Lee G.A., Zhao H.W., Chang Y.W., Lee C.J., Yang Y.C.S.H., Wu Y.C., Lin W.L., Liu Y.R., Ning D.S., Tseng S.H. (2022). KI Essence extract (a spleen-tonifying formula) promotes neurite outgrowth, alleviates oxidative stress and hypomyelination, and modulates microbiome in maternal immune activation offspring. Front. Pharmacol..

[31] Cuskelly A., Hoedt E.C., Harms L., Talley N.J., Tadros M.A., Keely S., Hodgson D.M. (2022). Neonatal immune challenge influences the microbiota and behaviour in a sexually dimorphic manner. Brain. Behav. Immun..

[32] Lin Y., Xie Z., Li Z., Yuan C., Zhang C., Li Y., Xie K., Wang K. (2024). The microbiota-gut-brain axis: A crucial immunomodulatory pathway for *Bifidobacterium animalis* subsp. lactis’ resilience against LPS treatment in neonatal rats. Int. J. Biol. Macromol..

[33] Romero-Miguel D., Casquero-Veiga M., Fernández J., Lamanna-Rama N., Gómez-Rangel V., Gálvez-Robleño C., Santa-Marta C., Villar C.J., Lombó F., Abalo R. (2023). Maternal Supplementation with N-Acetylcysteine Modulates the Microbiota-Gut-Brain Axis in Offspring of the Poly I:C Rat Model of Schizophrenia. Antioxidants.

[34] Tejkalová H., Jakob L., Kvasnová S., Klaschka J., Sechovcová H., Mrázek J., Páleníček T., Fliegerová K.O. (2023). The influence of antibiotic treatment on the behavior and gut microbiome of adult rats neonatally insulted with lipopolysaccharide. Heliyon.

[35] Tartaglione A.M., Villani A., Ajmone-Cat M.A., Minghetti L., Ricceri L., Pazienza V., De Simone R., Calamandrei G. (2022). Maternal immune activation induces autism-like changes in behavior, neuroinflammatory profile and gut microbiota in mouse offspring of both sexes. Transl. Psychiatry.

[36] Drobyshevsky A., Synowiec S., Goussakov I., Fabres R., Lu J., Caplan M. (2024). Intestinal microbiota modulates neuroinflammatory response and brain injury after neonatal hypoxia-ischemia. Gut Microbes.

[37] Prince N., Peralta Marzal L.N., Markidi A., Ahmed S., Adolfs Y., Pasterkamp R.J., Kumar H., Roeselers G., Garssen J., Kraneveld A.D. (2024). Prebiotic diet normalizes aberrant immune and behavioral phenotypes in a mouse model of autism spectrum disorder. Acta Pharmacol. Sin..

[38] Kers J.G., Saccenti E. (2022). The Power of Microbiome Studies: Some Considerations on Which Alpha and Beta Metrics to Use and How to Report Results. Front. Microbiol..

[39] Li Z., Zhou J., Liang H., Ye L., Lan L., Lu F., Wang Q., Lei T., Yang X., Cui P. (2022). Differences in Alpha Diversity of Gut Microbiota in Neurological Diseases. Front. Neurosci..

[40] Williams C.E., Hammer T.J., Williams C.L. (2024). Diversity alone does not reliably indicate the healthiness of an animal microbiome. ISME J..

[41] Wu Y., Gong Y., Zhang Y., Li S., Wang C., Yuan Y., Lv X., Liu Y., Chen F., Chen S. (2023). Comparative Analysis of Gut Microbiota from Rats Induced by Se Deficiency and T-2 Toxin. Nutrients.

[42] Zeng M.Y., Inohara N., Nuñez G. (2017). Mechanisms of inflammation-driven bacterial dysbiosis in the gut. Mucosal Immunol..

[43] Luca M., Chattipakorn S.C., Sriwichaiin S., Luca A. (2020). Cognitive-behavioural correlates of dysbiosis: A review. Int. J. Mol. Sci..

[44] Li Z., Lu G., Li Z., Wu B., Luo E., Qiu X., Guo J., Xia Z., Zheng C., Su Q. (2021). Altered Actinobacteria and Firmicutes Phylum Associated Epitopes in Patients With Parkinson’s Disease. Front. Immunol..

[45] Fang P., Kazmi S.A., Jameson K.G., Hsiao E.Y. (2020). The Microbiome as a Modifier of Neurodegenerative Disease Risk. Cell Host Microbe.

[46] Yong S.J., Tong T., Chew J., Lim W.L. (2020). Antidepressive Mechanisms of Probiotics and Their Therapeutic Potential. Front. Neurosci..

[47] Stojanov S., Berlec A., Štrukelj B. (2020). The influence of probiotics on the firmicutes/bacteroidetes ratio in the treatment of obesity and inflammatory bowel disease. Microorganisms.

[48] Maciel-Fiuza M.F., Muller G.C., Campos D.M.S., do Socorro Silva Costa P., Peruzzo J., Bonamigo R.R., Veit T., Vianna F.S.L. (2023). Role of gut microbiota in infectious and inflammatory diseases. Front. Microbiol..

[49] Silva Y.P., Bernardi A., Frozza R.L. (2020). The Role of Short-Chain Fatty Acids From Gut Microbiota in Gut-Brain Communication. Front. Endocrinol..

[50] Tang W., Meng Z., Li N., Liu Y., Li L., Chen D., Yang Y. (2021). Roles of Gut Microbiota in the Regulation of Hippocampal Plasticity, Inflammation, and Hippocampus-Dependent Behaviors. Front. Cell. Infect. Microbiol..

[51] Guzzetta K.E., Cryan J.F., O’Leary O.F. (2022). Microbiota-Gut-Brain Axis Regulation of Adult Hippocampal Neurogenesis. Brain Plast..

[52] Tillisch K., Mayer E.A., Gupta A., Gill Z., Brazeilles R., Le Nevé B., Van Hylckama Vlieg J.E.T., Guyonnet D., Derrien M., Labus J.S. (2017). Brain Structure and Response to Emotional Stimuli as Related to Gut Microbial Profiles in Healthy Women. Psychosom. Med..

[53] Cai X., Deng L., Ma X., Guo Y., Feng Z., Liu M., Guan Y., Huang Y., Deng J., Li H. (2020). Altered diversity and composition of gut microbiota in Wilson’s disease. Sci. Rep..

[54] Korteniemi J., Karlsson L., Aatsinki A. (2023). Systematic review: Autism spectrum disorder and the gut microbiota. Acta Psychiatr. Scand..

[55] Baldelli V., Scaldaferri F., Putignani L., Del Chierico F. (2021). The role of enterobacteriaceae in gut microbiota dysbiosis in inflammatory bowel diseases. Microorganisms.

[56] Hegde S., Lin Y.M., Golovko G., Khanipov K., Cong Y., Savidge T., Fofanov Y., Shi X.Z. (2018). Microbiota dysbiosis and its pathophysiological significance in bowel obstruction. Sci. Rep..

[57] Gryaznova M., Burakova I., Smirnova Y., Morozova P., Chirkin E., Gureev A., Mikhaylov E., Korneeva O., Syromyatnikov M. (2024). Effect of Probiotic Bacteria on the Gut Microbiome of Mice with Lipopolysaccharide-Induced Inflammation. Microorganisms.

[58] Dempsey E., Corr S.C. (2022). *Lactobacillus spp*. for Gastrointestinal Health: Current and Future Perspectives. Front. Immunol..

[59] Yoo J.Y., Groer M., Dutra S.V.O., Sarkar A., McSkimming D.I. (2020). Gut microbiota and immune system interactions. Microorganisms.

[60] Quansah M., David M.A., Martins R., El-Omar E., Aliberti S.M., Capunzo M., Jensen S.O., Tayebi M. (2025). The Beneficial Effects of *Lactobacillus* Strains on Gut Microbiome in Alzheimer’s Disease: A Systematic Review. Healthcare.

[61] Pan H., Yang S., Kulyar M.F., Ma H., Li K., Zhang L., Mo Q., Li J. (2025). *Lactobacillus* fermentum 016 Alleviates Mice Colitis by Modulating Oxidative Stress, Gut Microbiota, and Microbial Metabolism. Nutrients.

[62] Kim J., Lee H.J., Park S.K., Park J.H., Jeong H.R., Lee S., Lee H., Seol E., Hoe H.S. (2021). Donepezil regulates LPS and aβ-stimulated neuroinflammation through MAPK/NLRP3 inflammasome/STAT3 signaling. Int. J. Mol. Sci..

[63] Wallen Z.D., Appah M., Dean M.N., Sesler C.L., Factor S.A., Molho E., Zabetian C.P., Standaert D.G., Payami H. (2020). Characterizing dysbiosis of gut microbiome in PD: Evidence for overabundance of opportunistic pathogens. npj Park. Dis..

[64] Kullar R., Goldstein E.J.C., Johnson S., McFarland L.V. (2023). *Lactobacillus* Bacteremia and Probiotics: A Review. Microorganisms.

[65] Sribnick E.A., Popovich P.G., Hall M.W. (2022). Central nervous system injury—induced immune suppression. Neurosurg. Focus.

[66] Li B., Concepcion K., Meng X., Zhang L. (2017). Brain-immune interactions in perinatal hypoxic-ischemic brain injury. Prog. Neurobiol..

[67] Pariante C.M. (2003). Depression, Stressandthe Adrenalaxis. Neuroendocrinol. Briefings.

[68] Rau M., Rehman A., Dittrich M., Groen A.K., Hermanns H.M., Seyfried F., Beyersdorf N., Dandekar T., Rosenstiel P., Geier A. (2018). Fecal SCFAs and SCFA-producing bacteria in gut microbiome of human NAFLD as a putative link to systemic T-cell activation and advanced disease. United Eur. Gastroenterol. J..

[69] Chen Y., Liu Y., Wang Y., Chen X., Wang C., Chen X., Yuan X., Liu L., Yang J., Zhou X. (2022). Prevotellaceae produces butyrate to alleviate PD-1/PD-L1 inhibitor-related cardiotoxicity via PPARα-CYP4X1 axis in colonic macrophages. J. Exp. Clin. Cancer Res..

[70] Wang J., Chen W.D., Wang Y.D. (2020). The Relationship Between Gut Microbiota and Inflammatory Diseases: The Role of Macrophages. Front. Microbiol..

[71] Hasegawa S., Goto S., Tsuji H., Okuno T., Asahara T., Nomoto K., Shibata A., Fujisawa Y., Minato T., Okamoto A. (2015). Intestinal dysbiosis and lowered serum lipopolysaccharide-binding protein in Parkinson’s disease. PLoS ONE.

[72] Ferreira A.C., Freire M., Siqueira V., Ferreira C., Santos M.T. (2021). Brain Injury and Neuroinflammation of the Gut-Brain Axis in Subjects with Cerebral Palsy. Advancement and New Understanding in Brain Injury.

[73] Lyu J., Zhang X., Xiong S., Wu H., Han J., Xie Y., Qiu F., Yang Z., Huang C. (2024). Different care mode alter composition and function of gut microbiota in cerebral palsy children. Front. Pediatr..

[74] Möller B., Kollert F., Sculean A., Villiger P.M. (2020). Infectious Triggers in Periodontitis and the Gut in Rheumatoid Arthritis (RA): A Complex Story About Association and Causality. Front. Immunol..

[75] Huang C., Li Y., Feng X., Li D., Li X., Ouyang Q., Dai W., Wu G., Zhou Q., Wang P. (2019). Distinct Gut Microbiota Composition and Functional Category in Children With Cerebral Palsy and Epilepsy. Front. Pediatr..

