# Supplementary material for: Impact of Early-Life Brain Injury on Gut Microbiota Composition in Rodents: Systematic Review with Implications for Neurodevelopment
Source: Cells. 2025 Jul 11;14(14):1063. doi: 10.3390/cells14141063 (PMC12293283; doi:10.3390/cells14141063)
Supplement: Supplementary file 1 [file cells-14-01063-s001.zip › Supplementary Table S5.pdf]

This supplementary file presents the secondary outcomes of the systematic review entitled "Impact of Early-life Brain Injury on Gut Microbiota Composition in Rodents: Systematic Review with Implications for Neurodevelopment".

Table S5. Summary of secondary outcomes reported in the included studies.

Author and year	Brain-related outcomes	Intestinal alterations	Microbial metabolites	Inflammatory markers
Chen et al., 2025	- Activation of microglia and astrocytes in the hippocampal CA1 region of HI rats (ps<0.05).<br>- Decrease in the thickness of the postsynaptic density, expansion of the synaptic cleft, reduction in the density of dendritic spines and dendritic branches, nuclear shrinkage, neuronal loss, and reduced thickness in the hippocampal CA1 region of HI rats (ps<0.05).	- Worsened histopathological damages and higher histology scores in the colon in HI rats (p<0.05).<br>- Reduction in the colonic Occludin and ZO-1 expression in HI rats (ps<0.05).<br>- IL-17a and IL-22 increased in the HI group (ps<0.05).	Not assessed	- The serum concentrations of LPS, TNF-α, IL-6, and IL-1β were increased in the HI group (ps<0.05).
Chu et al., 2024	Not assessed	Not assessed	There was no statistically significant difference between the groups of interest.	- ↑IL-1β level in CP group on P28 compared with P14 (p<0.05).<br>- ↑IL-8 levels in CP group compared to Con (p<0.05).
Drobyshevsky et al., 2024	- Brain tissue: ↑TNFα, ↑IL2 and ↑TGFβ expressions in HI group compared to Sham (ps<0.05).<br>- ↑TLR2 and ↑TLR6 expressions in HI group compared to Sham (ps<0.05).	- Small intestine: villi density was decreased in HI group (p<0.05).	Not assessed	Not assessed
He et al., 2022	- An infarct volume (29.5 ± 2.10%) was observed in the injured brain hemisphere of HI rats<br>- HI induced neuronal injury in the cerebral cortex (neuronal shrinkage, nuclear chromatin condensation, and loss of intercellular connections).<br>- Hippocampus: disordered arrangement of neurons, unclear hierarchy, reduced	Not assessed	- Pathways downregulated in HI group: Butanoate metabolism, fatty acid biosynthesis, pantothenate and CoA biosynthesis, neuroactive ligand-receptor interaction, pentose phosphate pathway, and glycine, serine, and threonine metabolism (p<0.05).<br>- Pathway upregulated in HI group: HIF-1 signaling pathway (p<0.05).	Not assessed

	number of neurons, and partial nucleoplasmic shrinkage<br><br>- ↓SCFAs in the brain of HI group, including acetic acid ($p=0.040$) and butyric acid ($p<0.001$).<br><br>- Histone crotonylation was abundant in the brain and more altered in the HIBD group (acetylation $p = 0.041$, crotonylation $p<0.01$)<br><br>- ↓H3K9cr and ↑H3k18cr in the HI group ($ps<0.05$).<br><br>- ACADS expression was greater in the Sham group ($p < 0.05$)<br><br>- Several significantly differentially expressed genes between HI and Sham groups, including 465 upregulated and 482 downregulated genes. Gene Ontology (GO) terms indicated that enriched differentially expressed genes were related to neuron development, metabolic processes, and chromatin/histone modification.			
Huang et al., 2022	Not assessed	- The intestinal structure of pups exposed to LPS at first days of life were disordered; on P7, the intestinal mucosa structure was better than before.<br><br>- The Intestinal Injury Scores of the pups exposed to LPS were higher than that of the sham group at P0 ($p=0.045$) and P3 ($p=0.038$).<br><br>- ↓ZO-1 expression in the intestines of LPS rats at P0 ($p=0.005$) and P3 ($p=0.003$).	Not assessed	Not assessed
Jia et al., 2024	- ↑Nd content in the brain of ND rats ($p<0.05$).<br>- ↑BBB permeability in ND rats ($p<0.05$).<br>- ↓Occludin and ↓ZO-1 in the brains of ND rats ($ps<0.05$).	- The colonic tissue structure was abnormal in ND rats, accompanied by a small amount of	- ↓Acetic acid, ↓propionic acid, ↓butyric acid, ↑valeric acid (1% and 2%), ↑isovaleric acid (1%), and ↓isobutyric acid (1% and 2%) contents in ND groups ($p<0.05$).	Not assessed

	- The BBB became irregular, and the cerebral cortex became abnormal in the ND groups.	inflammatory cell infiltration.<br>- ↓Number of goblet cells in ND groups ($p<0.05$).<br>- ↑Thickness of the colonic mucus layer in ND group ($p<0.05$).		
Lee et al., 2021	- ↓MBP+ area in the MIA group's prefrontal cortex and thalamic nucleus ($p<0.05$).	Not assessed	Not assessed	Not assessed
Lee et al., 2022	- ↓MBP+ area in the prefrontal cortex and thalamic nucleus of MIA group ($p<0.05$).	Not assessed	Not assessed	Not assessed
Li et al., 2021	There was no statistically significant difference between the groups of interest	- PolyIC group exhibited distortions of gut morphology ($p <0.0001$) and severe cellular injury in the colons ($p =0.0486$)<br>- ↓ZO-1 ($p=0.0081$) and ↓CLDN ($p=0.0110$) levels in the colon tissue of PolyIC group.<br>- The colon tissue of PolyIC rats displayed elevated mRNA and protein expression levels for IL-1β and TNF- α (ps <0.05)	Not assessed	Not assessed
Lin et al., 2024	- ↑IL-6 and ↑TNFα levels in the hippocampus and cerebral cortex in the LPS group (ps<0.001).<br>- Neuronal cell structures in the hippocampus of LPS animals appeared blurred and disorganized, accompanied by decreased Nissl bodies and normal neurons in LPS.<br>- TLRs 2, 4 and 5 and the MYD88 exhibited activation in response to LPS stimulation in the hippocampus and cerebral cortex.<br>- Genes associated with nerve growth factor/brain-derived neurotrophic factors (IGF-1, NGF, VGF, BDNF, GDNF, NT3 and GAP43) were reduced activated in LPS group.	- ↑IL-6 and ↑TNFα levels in the colon of LPS group (ps<0.001).	- LPS disrupted the metabolic profile of the neonatal rat gut microbiota by inhibiting normal levels (21 metabolites down-regulated by LPS).	- ↑IL-6 and ↑TNFα serum levels in LPS rats (ps<0.001).
Ni et al., 2019	Not assessed	- ↑Glycolysis genes (Gck, Fas, Acc, Fatp, Fabp1, Dgat2, Gpat), in the livers of HI rats (ps<0.05)	- ↑functions relating to susceptibility to bacterial infections in the HI group (bacterial motility proteins, flagellar assembly, two component	Not assessed

		- HI led to a massive inflammatory cell infiltration in the colon tissue (p=0.018).<br>- ↓Secretion of mucin in the colon of HI rats (p<0.001).<br>- Tnf, Ifng, Sirt1 and Pgc1a were upregulated (ps<0.05) and Defb1, Defa8, and Muc3, were downregulated in the colon of HI rats (ps< 0.05).	systems, bacterial chemotaxis and secretion systems).<br><br>- ↓functions related to cellular processes and signaling in the HI group (membrane and intracellular structure, purine metabolism, other ion-coupled transporters, ion channels, chromosomes, other transporters, base excision repair, cell division and cell motility and secretion).<br><br>- metabolism functions were affected by the HI injury.<br><br>- ↓functions related to antibiotic synthesis in the HI group (streptomycin, penicillin, cephalosporin, butirosin and neomycin biosynthesis).	
Palanivelu et al., 2024	DTI:<br><br>- ↓Fractional Anisotropy in the ACC, thalamus and hippocampus on P21 and P49 (ps<0.05) and in the ACC, STR, TH and HIPP on P35 (p<0.05) in ASD rats compared to Con.<br>- ↑Mean Diffusivity in the ACC, thalamus, hippocampus and striatum on P21 and P49 (ps<0.05), and in the ACC, thalamus and HIPP on P35 (p<0.05) in ASD rats compared to Con.<br><br>- Higher density of GFAP-positive and Iba1-positive cells in ACC, hippocampus, striatum and thalamus regions of ASD rats (ps<0.05).	Not assessed	SCFA concentrations<br><br>- ↑Formate, ↓Acetate and ↓butyrate concentrations in the ASD group at P21, P35, and P49 (ps<0.05).	- ↑IL-1β, ↑IL-6, ↑IFN-γ, and ↑TNFα levels in ASD rats (with IL-1β and TNFα levels increasing progressively) (ps<0.05).
Prince et al., 2024	- ↑TMEM119 in the cerebellum of VPA animals (p<0.05).<br>- ↓Number of Tph2+ cells in the raphe nuclei of VPA animals (p<0.01).	- VPA mice displayed reduced levels of ZO-1 protein in the ileum compared to PBS (p<0.001).	**Relationship between microbial activity and differential taxa**<br><br>- A positive correlation was observed between: propionic acid and *Prevotella*, butyric acid and Bacteroidales, and acetic acid and Clostridiales. Peptococcaceae was also positively correlated with isobutyric acid, isovaleric acid, and valeric acid.<br><br>- A negative correlation was observed between: acetic acid and *Prevotella*, butyric acid and *Ruminococcus*.	- ↓Haptoglobin serum levels in VPA group (p<0.05).

Romero-Miguel et al., 2023	- The hippocampal volume was reduced in MIS versus the Con group ($p < 0.01$)	Not assessed	Not assessed	Not assessed
Tao et al., 2021	**-** The left-brain motor cortex and cingulate cortex of the CP group were destroyed by resecting the cortex and subcortical white matter	Not assessed	Not assessed	- ↑IL-6 and ↑TNF-α concentrations in the CP group ($p<0.01$)<br>- ↑CORT and ↑ACTH concentrations in the CP group ($p<0.01$)<br>- CP rats showed a higher fecal water content ($p<0.01$)
Tartaglione et al., 2022	**Hippocampus at P28**<br>- TNF-α, IL-6 and iNOS increased in Poly IC females.<br>- ↓BDNF and ↑Arg-1 in Poly IC mice (both sexes).<br>- Regardless of prenatal treatment, females expressed ↓TREM2 and ↑GFAP levels compared to males.<br><br>Cerebellum at P28<br>- TREM2, CD68 and GFAP increased in Poly IC females.<br>- ↑TNF-α in Poly IC mice (both sexes).<br>- Regardless of prenatal treatment, females expressed ↑iNOS and ↑Tmem119 levels compared to males.<br><br>**Hippocampus at P120**<br>- ↓Arg-1 in Poly IC mice (both sexes).<br>- Regardless of prenatal treatment, females expressed ↓TNF-α, ↓IL-6, ↓TGF-β, ↓TREM2, ↓Tmem119, ↓CD68, ↓GFAP levels compared to males.<br><br>Cerebellum at P120<br>- ↑TNF-α and ↑IL-6 in Poly IC mice (both sexes).<br>- ↑BDNF and ↓IL-1β in Poly IC females.<br>- ↑Tmem119 in Poly IC males.<br>- Regardless of prenatal treatment, females expressed ↑GFAP e ↓iNOS levels compared to males.	Not assessed	Not assessed	Not assessed
Tejkalová et al., 2023	Not assessed	Not assessed	Not assessed	Not assessed

Wei et al., 2025	- ↑LPS, ↑IL-6, ↑IL-1β, and ↑TNF-*α* levels in the hippocampus of HI animals (ps<0.05).<br>- ↑TLR4, ↑MyD88, ↑p-IκB*α* and ↑p-NF-κB p65 levels in the hippocampus of HI group (p<0.001).<br>- ↑Iba1+ and ↑TUNEL+ cells and ↓CA1 neurons in the hippocampus of HI group (p<0.001).<br>- Gene Ontology analysis: ↑DEGs in the signaling pathways related to inflammation and cytokine activity in the hippocampus of HI group.<br>- KEGG and GSEA: hippocampus of HI rats presented exacerbation of focal adhesion, tight junction, and the Toll-like receptor and NF-κB signaling pathways, and the insult impacted the signaling pathways associated with cytokine production, inflammatory responses, and tight junction integrity.	- The histological H&E scores were significantly higher in the colon of HI animals (p<0.001).<br>- ↓Occludin and ↓claudin-1 expression in the colon tissue of HI group (p<0.001).<br>- HI insult resulted in the leakage of pathogenic LPS in the feces (p<0.01)<br>- Gene Ontology analysis: ↑DEG in the signaling pathways related to inflammation and cytokine activity in the colon of HI group.<br>- KEGG and GSEA: hippocampus of HI rats presented exacerbation of focal adhesion, tight junction, and the Toll-like receptor and NF-κB signaling pathways, and the insult impacted the signaling pathways associated with cytokine production, inflammatory responses, and tight junction integrity.	Not assessed	- ↑LPS, ↑IL-6, ↑IL-1β, and ↑TNF-*α* levels in the serum of HI group (ps<0.05).<br>- The Spearman correlation analysis revealed a positive correlation between the abundance of *Enterobacteriaceae* and the levels of inflammatory cytokines, as well as histopathological damage<br>- HI insult resulted in the leakage of pathogenic LPS in the serum (p<0.001).
Yan et al., 2024	- Hypoxic rats exhibited lower brain weight (p < 0.05)<br>- Hypoxic rats exhibited more pronounced WMI, characterized by increased microglial activation and demyelination: ↑Iba1+ (p<0.05), ↓Luxol blue staining of myelin (p<0.01), ↓CC1+ oligodendrocytes (p<0.01), and ↓MBP (p<0.01).	Not assessed	Liquid chromatography tandem mass spectrometry (LC-MS).<br>- Chronic hypoxia resulted in 297 increased metabolites and 610 decreased (p<0.05).<br>- Significantly different metabolites were also enriched in the bile acid metabolism pathways.<br>- Cholic acid showed a strong positive correlation with *Bacteroides thetaiotaomicron* and *Parabacteroides distasonis* in the primary bile acid biosynthesis pathway (p<0.01).<br>- ↑Cholic acid levels in brain, serum and feces (p<0.05).	Not assessed
Zamudio-Flores et al., 2025	- TBI rats showed a smaller CEA/cell-body area ratio compared to Sham (p=0.008) in the cortex adjacent to the injury.	Not assessed	There was no statistically significant difference between the groups of interest.	Not assessed

	- In the ipsilateral hilus hippocampus subfield, TBI showed a smaller CEA/cell-body area ratio compared to Sham ($p<0.001$).	
