# Supplementary material for: Impact of Early-Life Brain Injury on Gut Microbiota Composition in Rodents: Systematic Review with Implications for Neurodevelopment
Source: Cells. 2025 Jul 11;14(14):1063. doi: 10.3390/cells14141063 (PMC12293283; doi:10.3390/cells14141063)
Supplement: Supplementary file 1 [file cells-14-01063-s001.zip › Supplementary Tables S1-S4.pdf]

This supplementary file presents the full search strategies used in each database for the systematic review entitled "Impact of Early-life Brain Injury on Gut Microbiota Composition in Rodents: Systematic Review with Implications for Neurodevelopment". The search was conducted in February 2025 across four databases: PubMed (Medline), Web of Science, Scopus, and Embase.

Table S1. PubMed Search Strategy

Component	Terms/Boolean operators
**Early Brain Injury**	Early Brain Injury OR Brain Injury OR Early Brain Injuries OR Brain Injuries OR Neonatal Brain Injury OR Early Brain Damage OR Brain Hemorrhage OR Traumatic Brain Injury OR Traumatic Brain Injuries OR Brain Trauma OR Brain Traumas OR Traumatic Encephalopathy OR Traumatic Encephalopathies OR Cerebral Palsy OR Cerebral Palsies OR Brain Hypoxia Ischemia OR Brain Hypoxia-Ischemia OR Brain Ischemia-Hypoxia OR Brain Ischemia Hypoxia OR Cerebral Ischemia-Hypoxia OR Cerebral Ischemia Hypoxia OR Cerebral Hypoxia-Ischemia OR Cerebral Hypoxia Ischemia OR Early Brain Ischemia OR Early Brain Hypoxia OR Ischemic Hypoxic Encephalopathy OR Hypoxic Ischemic Encephalopathy OR Neonatal Asphyxia OR Perinatal Asphyxia OR Maternal Inflammation OR Lipopolysaccharide Maternal Exposure OR Neuroinflammatory OR LPS Exposure OR Neuroinflammation
**AND**<br>**Microbiota**	Microbiota OR Microbiotas OR Microbial Community OR Microbial Communities OR Microbial Community Composition OR Microbial Community Compositions OR Microbiome OR Microbiomes OR Microbial Community Structure OR Microbial Community Structures OR Gastrointestinal Microbiomes OR Gastrointestinal Microbial Community OR Gastrointestinal Microbial Communities OR Gut Microbiome OR Gut Microbiomes OR Gut Microflora OR Gastrointestinal Microflora OR Gastrointestinal Flora OR Gut Flora OR Gastrointestinal Microbiota OR Gastrointestinal Microbiotas OR Gut Microbiota OR Gut Microbiotas OR

	Intestinal Microbiome OR Intestinal Microbiomes OR Intestinal Flora OR Intestinal Microbiota OR Intestinal Microbiotas OR Intestinal Microflora OR Gastric Microbiome OR Gastric Microbiomes OR Gut Dysbiosis OR Gut-Brain Axis OR Intestinal Dysbiosis
**AND**<br>**Animals (filter)**	animal experimentation[MeSH] OR models, animal[MeSH] OR Animals[Mesh:noexp] OR animal population groups [MeSH] OR rat[tiab] OR rats[tiab] OR animal[tiab] OR animals[tiab] OR mice[tiab] OR in vivo[tiab] OR mouse[tiab]

Table S2. Scopus Search Strategy

**Component**	**Terms/Boolean operators**
**Early Brain Injury**	"Early Brain Injury" OR "Brain Injury" OR "Early Brain Injuries" OR "Brain Injuries" OR "Neonatal Brain Injury" OR "Early Brain Damage" OR "Brain Hemorrhage" OR "Traumatic Brain Injury" OR "Traumatic Brain Injuries" OR "Brain Trauma" OR "Brain Traumas" OR "Traumatic Encephalopathy" OR "Traumatic Encephalopathies" OR "Cerebral Palsy" OR "Cerebral Palsies" OR "Brain Hypoxia Ischemia" OR "Brain Hypoxia-Ischemia" OR "Brain Ischemia-Hypoxia" OR "Brain Ischemia Hypoxia" OR "Cerebral Ischemia-Hypoxia" OR "Cerebral Ischemia Hypoxia" OR "Cerebral Hypoxia-Ischemia" OR "Cerebral Hypoxia Ischemia" OR "Early Brain Ischemia" OR "Early Brain Hypoxia" OR "Ischemic Hypoxic Encephalopathy" OR "Hypoxic Ischemic Encephalopathy" OR "Neonatal Asphyxia" OR "Perinatal Asphyxia" OR "Lipopolysaccharide Maternal Exposure" OR "Neuroinflammatory" OR "LPS Exposure" OR "Neuroinflammation"
**AND**<br>**Microbiota**	"Microbiota" OR "Microbiotas" OR "Microbial Community" OR "Microbial Communities" OR "Microbial Community Composition" OR

	"Microbial Community Compositions" OR "Microbiome OR Microbiomes" OR "Microbial Community Structure" OR "Microbial Community Structures" OR "Gastrointestinal Microbiomes" OR "Gastrointestinal Microbial Community" OR "Gastrointestinal Microbial Communities" OR "Gut Microbiome" OR "Gut Microbiomes" OR "Gut Microflora" OR "Gastrointestinal Microflora" OR "Gastrointestinal Flora" OR "Gut Flora" OR "Gastrointestinal Microbiota" OR "Gastrointestinal Microbiotas" OR "Gut Microbiota" OR "Gut Microbiotas" OR "Intestinal Microbiome" OR "Intestinal Microbiomes" OR "Intestinal Flora" OR "Intestinal Microbiota" OR "Intestinal Microbiotas" OR "Intestinal Microflora" OR "Gastric Microbiome" OR "Gastric Microbiomes" OR "Gut Dysbiosis" OR "Gut-Brain Axis" OR "Intestinal Dysbiosis"

Table S3. Web of Science Search Strategy

Component	Terms/Boolean operators
**Early Brain Injury**	Early Brain Injury OR Neonatal Brain Injury OR Early Brain Damage OR Traumatic Brain Injury OR Traumatic Encephalopathy OR Cerebral Palsy OR Brain Hypoxia Ischemia OR Brain Hypoxia-Ischemia OR Cerebral Ischemia-Hypoxia OR Cerebral Ischemia Hypoxia OR Cerebral Hypoxia-Ischemia OR Cerebral Hypoxia Ischemia OR Ischemic Hypoxic Encephalopathy OR Hypoxic Ischemic Encephalopathy OR Neonatal Asphyxia OR Perinatal Asphyxia OR Lipopolysaccharide Maternal Exposure OR Neuroinflammatory OR LPS Exposure OR Neuroinflammation
**AND**<br>**Microbiota**	Microbiotas OR Microbial Community OR Microbiome OR Microbiomes OR Microbial Community Structure OR Gastrointestinal Microbial Community OR Gut Microbiome OR Gastrointestinal Microbiota OR Gut

	Microbiota OR Intestinal Microbiome OR Intestinal Microbiota OR Gut Dysbiosis OR Gut-Brain Axis OR Intestinal Dysbiosis
**AND**<br>**Animals (filter)**	Rodents OR Rodent OR Rat OR Rats OR Mice OR Mouse

Table S4. EMBASE Search Strategy

**Component**	**Terms/Boolean operators**
**Early Brain Injury**	Early Brain Injury OR Neonatal Brain Injury OR Early Brain Damage OR Traumatic Brain Injury OR Traumatic Encephalopathy OR Cerebral Palsy OR Brain Hypoxia Ischemia OR Brain Hypoxia-Ischemia OR Cerebral Ischemia-Hypoxia OR Cerebral Ischemia Hypoxia OR Cerebral Hypoxia-Ischemia OR Cerebral Hypoxia Ischemia OR Ischemic Hypoxic Encephalopathy OR Hypoxic Ischemic Encephalopathy OR Neonatal Asphyxia OR Perinatal Asphyxia OR Lipopolysaccharide Maternal Exposure OR Neuroinflammatory OR LPS Exposure OR Neuroinflammation
**AND**<br>**Microbiota**	Microbiotas OR Microbial Community OR Microbiome OR Microbiomes OR Microbial Community Structure OR Gastrointestinal Microbial Community OR Gut Microbiome OR Gastrointestinal Microbiota OR Gut Microbiota OR Intestinal Microbiome OR Intestinal Microbiota OR Gut Dysbiosis OR Gut-Brain Axis OR Intestinal Dysbiosis
**AND**<br>**Animals (filter)**	rat OR rats OR animal OR animals OR mice OR "in vivo" OR mouse
